# Genome-wide analyses reveal the IRE1a-XBP1 pathway promotes T helper cell differentiation by resolving secretory stress and accelerating proliferation

**DOI:** 10.1186/s13073-018-0589-3

**Published:** 2018-10-24

**Authors:** Jhuma Pramanik, Xi Chen, Gozde Kar, Johan Henriksson, Tomás Gomes, Jong-Eun Park, Kedar Natarajan, Kerstin B. Meyer, Zhichao Miao, Andrew N. J. McKenzie, Bidesh Mahata, Sarah A. Teichmann

**Affiliations:** 1 Wellcome Sanger Institute, Wellcome Genome Campus, Hinxton, Cambridge, CB10 1SA UK; 2 EMBL-European Bioinformatics Institute, Wellcome Genome Campus, Hinxton, Cambridge, CB10 1SD UK; 3 MRC Laboratory of Molecular Biology, Cambridge Biomedical Campus, Francis Crick Avenue, Cambridge, CB2 0QH UK; 4 Theory of Condensed Matter, Cavendish Laboratory, 19 JJ Thomson Ave, Cambridge, CB3 0HE UK

**Keywords:** IRE1a-XBP1 pathway, IRE1a, XBP1, T helper cell activation, Genome-wide XBP1 chromatin occupancy, Th2 lymphocyte proliferation, XBP1 ChIP-seq, RNA-seq, Th2 transcriptome

## Abstract

**Background:**

The IRE1a-XBP1 pathway is a conserved adaptive mediator of the unfolded protein response. The pathway is indispensable for the development of secretory cells by facilitating protein folding and enhancing secretory capacity. In the immune system, it is known to function in dendritic cells, plasma cells, and eosinophil development and differentiation, while its role in T helper cell is unexplored. Here, we investigated the role of the IRE1a-XBP1 pathway in regulating activation and differentiation of type-2 T helper cell (Th2), a major T helper cell type involved in allergy, asthma, helminth infection, pregnancy, and tumor immunosuppression.

**Methods:**

We perturbed the IRE1a-XBP1 pathway and interrogated its role in Th2 cell differentiation. We performed genome-wide transcriptomic analysis of differential gene expression to reveal IRE1a-XBP1 pathway-regulated genes and predict their biological role. To identify direct target genes of XBP1 and define XBP1’s regulatory network, we performed XBP1 ChIPmentation (ChIP-seq). We validated our predictions by flow cytometry, ELISA, and qPCR. We also used a fluorescent ubiquitin cell cycle indicator mouse to demonstrate the role of XBP1 in the cell cycle.

**Results:**

We show that Th2 lymphocytes induce the IRE1a-XBP1 pathway during in vitro and in vivo activation. Genome-wide transcriptomic analysis of differential gene expression by perturbing the IRE1a-XBP1 pathway reveals XBP1-controlled genes and biological pathways. Performing XBP1 ChIPmentation (ChIP-seq) and integrating with transcriptomic data, we identify XBP1-controlled direct target genes and its transcriptional regulatory network. We observed that the IRE1a-XBP1 pathway controls cytokine secretion and the expression of two Th2 signature cytokines, IL13 and IL5. We also discovered that the IRE1a-XBP1 pathway facilitates activation-dependent Th2 cell proliferation by facilitating cell cycle progression through S and G2/M phase.

**Conclusions:**

We confirm and detail the critical role of the IRE1a-XBP1 pathway during Th2 lymphocyte activation in regulating cytokine expression, secretion, and cell proliferation. Our high-quality genome-wide XBP1 ChIP and gene expression data provide a rich resource for investigating XBP1-regulated genes. We provide a browsable online database available at http://data.teichlab.org.

**Electronic supplementary material:**

The online version of this article (10.1186/s13073-018-0589-3) contains supplementary material, which is available to authorized users.

## Background

**Fig. 1:**
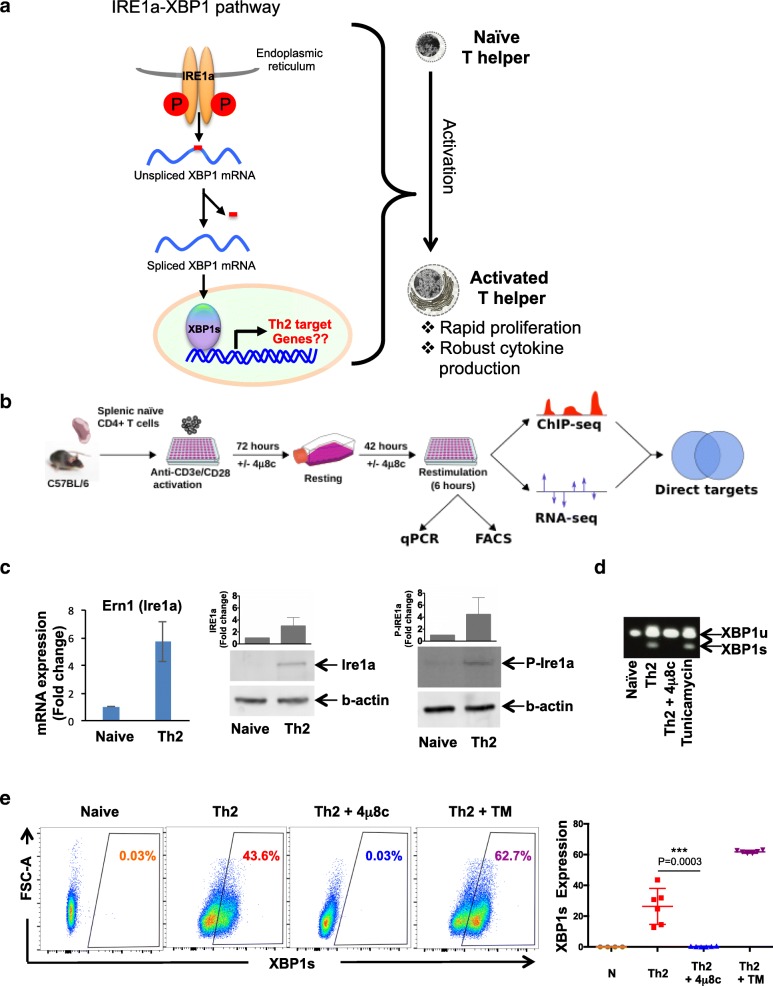
T helper cells upregulate the IRE1a-XBP1 pathway during activation. **a** Schematic representation of the hypothesis. In this study, we are asking what role does the IRE1a-XBP1 pathway play during T helper cell activation. T helper cell activation is a dramatic transformation from a quiescent cell state to a rapidly proliferative and highly protein productive/secretive cellular state. **b** Overview of the experiment. Splenic naïve T cells were purified by negative selection and activated in anti-CD3e/C28 antibody-coated plates under Th2 differentiation conditions (i.e., in the presence of anti-IFNγ neutralizing antibody, IL2, and IL4) for 72 h, rested for 42 h, and restimulated on anti-CD3e/CD28 antibody-coated plate. Restimulated Th2 cells were used in RNA sequencing, ChIPmentation (ChIP sequencing), Western blot, qPCR, and flow cytometry. To perturb IRE1a-XBP1 pathway, we used 15-μM 4μ8c that specifically blocks the pathway by inhibiting IRE1a endonuclease activity. The drug was added to the culture media at the beginning of the culture and during passage from the activation plate to the resting plate. **c** Naïve T helper cells and in vitro differentiated Th2 lymphocytes were analyzed for IRE1a mRNA expression by qRT-PCR (left panel), protein expression by Western blot (middle panel), and phosphorylated IRE1a (P-IRE1a) by Western blot (right panel). The density of Western blot bands from five independent experiments of IRE1a and three independent experiments of phospho-IRE1a were measured and displayed on top of each Western blot panel. **d** Naïve T cells were cultured under Th2 differentiation conditions in the presence or absence of IRE1a inhibitor (4μ8c). In vitro reactivated Th2 lymphocytes were analyzed by RT-PCR using a pair of primers that discriminate the cDNA derived from spliced and unspliced form of XBP1 mRNA. Tunicamycin-treated Th2 cells were used as a positive control. **e** Naïve T helper cells (N) and in vitro differentiated and restimulated Th2 cells (differentiated in the presence or absence of 4μ8c) were stained with fluorescent dye-conjugated anti-XBP1s-specific antibody and analyzed by flow cytometry. Gating: singlets > live cells > XBP1s. One representative FACS profile is displayed (left panel), and the graph containing all results (*n* = 5) is shown in the “right panel”. Tunicamycin-treated Th2 cells were used as a positive control

T helper (Th) cells (CD4^+^ T cells) are central to the adaptive immune response and immune tolerance and potentiate innate immune response pathways [1, 2]. These cells are key players in infections, allergies, auto-immunity, and anti-tumor immune responses. Depending upon the immunogen or allergen (e.g., infection, commensal microorganism, or self-antigen), naive T helper cells become activated, proliferate, and are able to differentiate into several subtypes, such as Th1, Th2, Th17, and regulatory T cell (Treg). This Th subtype classification is based on their differential expression of cytokines and key lineage-specific transcription factors [2, 3]. Th2 lymphocytes secrete the characteristic cytokines IL4, IL5, IL10, and IL13. These secretory cells are involved in worm parasite expulsion, exaggerate allergies and asthma, potentiate pregnancy [4], and suppress anti-tumor immunity [5]. Transcription factors that are involved in differential production and regulation of cytokine genes, for example GATA3 in Th2, are well studied. However, cytokine gene expression is only one aspect of the T helper cell differentiation process. The ability to rapidly proliferate is another key attribute of T helper lymphocytes (Fig. 1a), and the full regulatory circuitry controlling these processes is still incompletely understood.

Proliferation is required for clonal expansion, which forms the basis of the adaptive immune response [6, 7]. The Gata3/RuvB-like protein 2 (Ruvbl2) complex was shown to be a key regulator of Th2 cell proliferation [8], and several other transcription factors, such as Stat6, are implicated in the regulatory circuitry controlling T helper cell proliferation and differentiation. Additional transcription factors are likely to be involved in regulating this highly organized, complex process.

At the cell biological level, to synthesize, fold, and secrete proteins, including cytokines, activated T helper cells need to contain a well-differentiated endoplasmic reticulum (ER) and protein secretory machinery. It is an open question how activated T helper cells meet this protein folding and secretory demand. Secretory cells (e.g., pancreatic β-cell, acinar cells) address this challenge by upregulating the unfolded protein response (UPR) pathway triggered by the accumulation of unfolded proteins in the endoplasmic reticulum (ER) [9–11]. Three ER membrane-resident sensors, the endonuclease IRE1a (encoded by *ERN1* gene), the kinase PERK, and the cleavable precursor of the transcription factor ATF6, coordinate the process. Among these three, the IRE1a-XBP1 pathway is the most evolutionary conserved pathway (Fig. 1a) [12, 13]. During ER stress, the kinase, IRE1a, oligomerizes, autophosphorylates, and uses its endoribonuclease activity to splice a 26-nucleotide fragment from the unspliced XBP1 mRNA (XBP1u). This then results in the functional spliced form of the transcription factor XBP1 (XBP1s) [14]. XBP1s regulates the expression of numerous target genes involved in ER biogenesis. Its role has been studied in secretory cells, such as pancreatic acinar cells, plasma cells, and dendritic cells (DCs). In these cell types, XBP1 occupies chromatin and controls gene expression in a cell-type-specific manner [15]. This suggests that XBP1 may play a role in diverse cell types. Therefore, we set out to investigate its specific function in CD4^+^ T lymphocytes (Fig. 1a).

The role of the IRE1a-XBP1 pathway in immunity and inflammation is now emerging [16–20]. The pathway has been described in dendritic cells, plasma cells, CD8^+^ T cells, and eosinophil development and differentiation [21–26]. Interestingly, it has been reported recently that the pathway causes cancer-associated immune suppression by causing dendritic cell dysfunction [27]. The pathway is also involved in alternative activation of macrophages and in obesity [28]. Together, these reports suggest that the XBP1 transcription factor can contribute to a wide range of biological processes. IRE1a inhibitors (e.g., 4μ8c) have been proposed as a treatment of cancer, by reinstating cancer immunity and eosinophilia by inhibiting eosinophil differentiation [21, 27, 29, 30]. Here, we test the role of the XBP1 transcription factor in regulating T helper cell activation through inhibition of the IRE1a-XBP1 pathway by the small molecule inhibitor 4μ8c.

Using genome-wide approaches, integrating transcriptomic and XBP1 chromatin occupancy data, we elucidate the regulatory circuitry governed by the IRE1a-XBP1 pathway in Th2 lymphocytes. We found that the pathway observed in other cells is conserved in T helper cells in terms of secretory stress adaptation. Further, we show that XBP1 regulates genes that control diverse facets of Th2 cell physiology. In addition to resolving protein folding and secretory stress, it accelerates cell proliferation and controls cytokine synthesis and secretion.

Our data provide a rich resource for investigating XBP1-regulated genes with genome-wide chromatin occupancy and expression, with a browsable online database at http://data.teichlab.org.

## Methods

### Materials

CD4^+^CD62L^+^ T Cell Isolation Kit II, mouse (Miltenyi Biotec, 130-093-227); Naive CD4^+^ T Cell Isolation Kit, mouse (Miltenyi Biotec, 130-104-453); FITC BrdU Flow Kit (BD Pharmingen, 51-2354AK); LIVE/DEAD™ Fixable Violet Dead Cell Stain Kit (Molecular probes, L34955); CellTrace™ Violet Cell Proliferation Kit (Molecular probes, C34571); Mouse IL-13 ELISA Ready-SET-Go Kit (eBioscience, 88-7137-22); Mouse IL-4 ELISA Ready-SET-Go Kit (eBioscience, 88-7044-88); Mouse IL-5 ELISA (BD Biosciences, 555236); PE Mouse anti-XBP1S Clone Q3-695 (BD Pharmingen, 562642); XBP1 (M-186)X- (Santa cruz, Sc 7160x); IL5-PE (BD Pharmingen, 554395); IL4-APC, Clone 11B11 (eBioscience, 17-7041-82); IL13-AF488, Clone eBio3A (eBioscience, 53-7133-82); IFNγ-Per CP Cy5.5, Clone XMG1.2 (eBioscience, 45-7311-82); FACS Staining buffer (eBioscience, 00-4222-26); IC Fixation buffer (eBioscience, 00-8222-49); Fixation/Permeabilization Diluent (eBioscience, 00-5223-56); Fixation/Permeabilization concentrate (eBioscience, 00-5123-43); Permeabilization buffer (eBioscience, 00-8333-56); SV Total RNA Isolation System (Promega, Z3101); Transcriptor High Fidelity cDNA Synthesis kit (Roche, 05081955001); SYBR™ Select Master Mix (Applied Biosystems, 4472908); Western blot antibodies: IRE1α (14C10) Rabbit mAb (Cell Signaling, #3294), IRE1 alpha [p Ser724] Antibody (Novus biologicals,NB100-2323).

### Mice

The mice (C57BL/6, IL13-eGFP reporter, IL4-eGFP reporter, and FUCCI) were maintained under specific pathogen-free conditions at the Wellcome Trust Genome Campus Research Support Facility (Cambridge, UK) and were used at 6–12 weeks of age. We generated a transgenic FUCCI mouse, similar to published FUCCI strain [31].

### T helper cell culture

Splenic naive T helper cells were purified with the CD4^+^CD62L^+^T Cell Isolation Kit II (Miltenyi Biotec) and polarized in vitro toward differentiated Th2 subtype as described before in [32]. In brief, naive cells were seeded into anti-CD3e (2 μg/ml, clone 145-2C11, eBioscience) and anti-CD28 (5 μg/ml, clone 37.51, eBioscience) antibody coated 96-well round bottom plates. The medium contained the following cytokines and/or antibodies for Th2 subtype: recombinant murine IL-2 (10 ng/ml, R&D Systems), recombinant murine IL-4 (10 ng/ml, R&D Systems), and neutralizing anti-IFN-g (5 μg/ml, cloneXMG1.2eBioscience). The cells were removed from the activation plate on day 4 (72 h). Th2 cells were cultured for another 2 days in the absence of anti-CD3 and CD28 stimulation. Then, cells were restimulated by anti-CD3e/CD28-coated plate for 6 h. For flow cytometric detection, cells were treated with monensin (2 μM, eBioscience) for the last 3 h.

#### 4μ8c treatment

4μ8c (final concentration 15 μM) was added to the culture media at the beginning of the culture, and with the fresh culture media when cells were transferred from the activation plate to the resting plate.

### Reverse transcription quantitative PCR (RT-qPCR*)*

Total RNA was isolated from two million cells by SV total RNA isolation kit (Promega). cDNA was prepared by annealing 500 ng RNA with oligo dT as per the manufacturer’s instructions (Transcriptor High Fidelity cDNA Synthesis kit, Roche). The cDNA samples were diluted 10 times with H20. Two microliters of cDNA was used in 12 μl qPCR reactions with appropriate primers and SYBR Green PCR Master Mix (Applied Biosystems). Experiments were performed at least three times and data represent mean values ± standard deviation. For XBP1, mRNA was amplified by PCR and products were separated by electrophoresis through a 2.5% agarose gel and visualized by ethidium bromide staining. The primer list is provided below:

- IL4-F: 5′-AACTCCATGCTTGAAGAAGAACTC-3′
- IL4-R: 5′-CCAGGAAGTCTTTCAGTGATGTG-3′
- IL13-F: 5′-CCTGGCTCTTGCTTGCCTT-3′
- IL13-R: 5′-GGTCTTGTGTGATGTTGCTCA-3′
- IL5-F: 5′-GCAATGAGACGATGAGGCTTC-3′
- IL5-R: 5′-CCCCTGAAAGATTTCTCCAATG-3′
- ERN1-F: 5′-ACACCGACCACCGTATCTCA-3′
- ERN1-R: 5′-CTCAGGATAATGGTAGCCATGTC-3′
- XBP1-F: 5′-ACACGCTTGGGAATGGACAC-3′
- XBP1-R: 5′-CCATGGGAAGATGTTCTGGG-3′
- RPLP0-F: 5′-CACTGGTCTAGGACCCGAGAA-3′
- RPLP0-R: 5′-GGTGCCTCTGGAGATTTTCG-3′
- XBP1s-F: 5′-ACACGCTTGGGAATGGACAC-3′
- XBP1s-R: 5′-GTGTCAGAGTCCATGGGA-3′

### ELISA

IL13, IL4, and IL5 concentration in the Th2 culture supernatants were quantified using ELISA kit following the manufacturer’s instruction (see the “Materials” section for the kit specification).

### Flow cytometry

In worm infection mouse experiments, splenocytes were prepared on day 7 post-infection from *Nippostrongylus brasiliensis* infected or control uninfected mice, stained with anti-CD3e, anti-CD4 (eBioscience), and XBP1s-PE (BD Pharmingen) antibodies following the mouse regulatory T cell staining kit protocol (eBioscience), and were measured by flow cytometry on a Fortessa (BD Biosciences) using FACSDiva. The data were analyzed by the FlowJo software. For in vitro Th cell experiments, staining was performed following eBioscience intracellular staining protocol for cytokines and nuclear staining/transcription factor staining protocol for XBP1 transcription factor using eBioscience reagents and kit protocol. The following antibodies were fluorescent dye-conjugated primary antibodies: IL-4, IL-13, IL-5, CD4 and IFNγ (eBioscience), and XBP1s (BD Pharmingen). Stained cells were analyzed on a Fortessa (BD Biosciences) using FACSDiva and FlowJo software. CompBeads (BD Biosciences) were used for compensation where distinct positively stained populations were unavailable.

### Cell proliferation assay

Naive Th cells were stained with CellTrace Violet following the CellTrace Violet Cell Proliferation Kit (Invitrogen) protocol and cultured under activation-differentiation conditions for Th2 as described previously, in the presence or absence of 15 μM 4μ8c for 4 days. Flow cytometry was performed using a BD Fortessa and data analysis with FlowJo software.

### *N. brasiliensis* infection and splenocyte preparation

C57BL/6 female mice were subcutaneously injected with 100 μl (300/500 live third stage *N. brasiliensis* larvae per dose). Spleen was taken from infected mice 7 days after infection. Cells were isolated from spleen by smashing the tissue through a 70-μm cell strainer and suspended in RBC lysis buffer (eBioscience). Single-cell suspensions of splenocytes were then stained following FACS staining protocol.

### Analysis of bulk RNA-sequencing data

For each sample, reads were mapped to the *Mus musculus* genome (GRCm38) using GSNAP with default parameters [33]. Uniquely mapped reads to the genome were counted using htseq-count (http://htseq.readthedocs.io/) and normalized with size factors calculated by DESeq2 [34]. Differentially expressed genes across conditions were identified using DESeq2 with an adjusted *p* value cutoff < 0.05.

### XBP1 ChIPmentation

In vitro differentiated and reactivated Th2 cells were used in ChIP. Two independent biological replicates were performed. Twenty million cells from each sample were crosslinked in 1% HCHO (prepared in 1X DPBS) at room temperature for 10 min, and HCHO was quenched by the addition of glycine at a final concentration of 0.125 M. Cells were pelleted at 4 °C at 2000×*g*, washed with ice-cold 1X DPBS twice, and snapped frozen in liquid nitrogen. The cell pellets were stored in − 80 °C until the experiments were performed. ChIPmentation was performed according to the version 1.0 of the published protocol [35] with some modifications at the ChIP stage.

Briefly, cell pellets were thawed on ice and lysed in 300 μl ChIP Lysis Buffer I (50 mM HEPES.KOH, pH 7.5, 140 mM NaCl, 1 mM EDTA, pH 8.0, 10% Glycerol, 0.5% NP-40, 0.25% Triton X-100) on ice for 10 min. Then, cells were pelleted at 4 °C at 2000×*g* for 5 min, washed by 300 μl ChIP Lysis Buffer II (10 mM Tris-Cl, pH 8.0, 200 mM NaCl, 1 mM EDTA, pH 8.0, 0.5 mM EGTA, pH 8.0), and pelleted again at 4 °C at 2000×*g* for 5 min. Nuclei were resuspended in 300 μl ChIP Lysis Buffer III (10 mM Tris-Cl, pH 8.0, 100 mM NaCl, 1 mM EDTA, 0.5 mM EGTA, 0.1% sodium deoxycholate, 0.5% *N*-lauroylsarcosine). Chromatin was sonicated using Bioruptor Pico (Diagenode) with 30 s ON/30 s OFF for 3 cycles. Thirty microliter 10% Triton X-100 were added into each sonicated chromatin, and insoluble chromatin was pelleted at 16,100×*g* at 4 °C for 10 min. One microliter supernatant was taken as input control. The rest of the supernatant was incubated with 10 μl Protein A Dynabeads (Invitrogen) pre-bound with 1 μg XBP1 antibody (XBP1 (M-186)X - Santa cruz), in a rotating platform in a cold room overnight. Each immunoprecipitation (IP) was washed with 500 μl RIPA Buffer (50 mM HEPES.KOH, pH 7.5, 500 mM LiCl, 1 mM EDTA, 1% NP-40, 0.7% Sodium Deoxycholate, check components) for three times. Then, each IP was washed with 500 μl 10 mM Tris, pH 8.0 twice, and resuspended in 30 μl tagmentation reaction mix (10 mM Tris.Cl, pH 8.0, 5 mM Mg2Cl, 1 μl TDE1 (Nextera)). Then, the tagmentation reaction was put on a thermomixer at 37 °C for 10 min at 800 rpm shaking. After the tagmentation reaction, each IP was washed sequentially with 500 μl RIPA Buffer twice, and 1X TE NaCl (10 mM Tris.Cl, pH 8.0, 1 mM EDTA, pH 8.0, 50 mM NaCl) once. Elution and reverse crosslinking was done by resuspending the beads with 100 μl ChIP Elution Buffer (50 mM Tris.Cl, pH 8.0, 10 mM EDTA, pH 8.0, 1% SDS) on a thermomixer at 65 °C overnight, 1400 rpm. DNA was purified by MinElute PCR Purification Kit (QIAGEN, cat no. 28004) and eluted in 12.5 μl Buffer EB (QIAGEN kit, cat no 28004), which yielded ~ 10 μl ChIPed DNA.

The library preparation reactions contained the following:

Ten-microliter purified DNA (from above), 2.5-μl PCR Primer Cocktails (Nextera DNA Library Preparation Kit, Illumina Cat no. FC-121-1030), 2.5 μl N5xx (Nextera Index Kit, Illumina cat no. FC-121-1012), 2.5 μl N7xx (Nextera Index Kit, Illumina cat no. FC-121-1012), 7.5 μl NPM PCR Master Mix (Nextera DNA Library Preparation Kit, Illumina cat no. FC-121-1030). PCR was set up as follows: 72 °C, 5 min; 98 °C, 2 min; [98 °C, 10 s, 63 °C, 30 s, 72 °C, 20 s] × 12; 10 °C hold. The amplified libraries were purified by double AmpureXP beads purification: first with 0.5X bead ratio, keep supernatant, second with 1.4X bead ratio, keep bound DNA. Elution was done in 20 μl Buffer EB (QIAGEN). One microliter of library was run on an Agilent Bioanalyzer to see the size distribution. Sequencing was done on an Illumina Hiseq2000 platform using the v4 chemistry (75 bp PE).

### ChIPmentation analysis

The reads were first trimmed using Trimmomatic 0.3664 with settings ILLUMINACLIP:NexteraPE-PE.fa:2:30:10 LEADING:3 TRAILING:3 SLIDINGWINDOW:4:15 MINLEN:30. Peaks were then called using MACS265, merged over time, and annotated using HOMER66.

The quality of the peaks was assessed using the two available replicates of XBP1.

#### Inferred regulatory cascade of XBP1

Transcription factors were obtained from the AnimalTFDB 2.0 [36] and were defined as targets of XBP1 if they were intersected by a ChIPmentation peak and differentially expressed between Th2 (control) and 4μ8c-treated Th2. Genes were defined as targeted by these transcription factors if in the STRING version 10 database [37], they had an “expression” mode of interaction with a score greater than 200 with these transcription factors in mouse, and were differentially expressed between Th2 (control) and 4μ8c-treated Th2.

### XBP1s overexpression and comparison with drug treatment

#### XBP1s cloning

To generate an pMSCV-XBP1s-IRES-mCherry construct, Flag-XBP1s was amplified from Flag-XBP1s-pcDNA5/FRT/TO (gift from Prof. David Ron) by PCR (F primer: cgccggaattcagatcttacgtagctagcgCAAATGGACTACAAAGACGA, R primer: gcggaattgatcccgctcgagcaattggTTAGACACTAATCAGCTGGG). This Flag-XBP1s fragment was integrated with pMSCV-IRES-mCherry fragment of pMSCV-IRES-mCherry FP (Addgene #52114, gift from prof. Dario Vignali) cut by bamHI.

#### Viral transduction

Virus was produced following the procedure as described in our previous publication (Henriksson et al. 2017, doi: 10.1101/196022). Briefly, platE cells were grown in Advanced DMEM with FBS, pen-strep (PS) and l-glutamine. A mix of 1-μg pCL-Eco (Addgene #12371, gift from Inder Verma, [38]), 1-μg retroviral plasmid, and 2-μl PLUS were mixed in 0.5 ml OptiMEM. The mix was vortexed and incubated for 2 min at room temperature. Six-microliter Lipofectamin LTX was added; the mix pipetted up and down and incubated for 30 min at room temperature. The mix was added to one well of a six-well plate, containing 80–90% cells and freshly replaced 1.5-ml OptiMEM. Five hours later, the media was replaced with 2-ml Advanced DMEM. The morning after the media was again replaced with 1.5-ml Advanced DMEM. Forty-eight hours after this replacement, the virus was harvested. The supernatant was filtered by centrifugation (1000*g*, 4 °C, 10 min) and stored at 4 °C overnight.

Naive CD4^+^ T cells were purified by negative selection using MACS as described above and plated on anti-CD3/CD28-coated plates under Th2 differentiation condition with or without 15 μM 4μ8c on the same day as the virus harvest. The next day, 40-μl IMDM + 160-μl virus supernatant was added into each well of a 96-well plate (round-bottom). To this mix, we added 55 μM βME (2-ME), 8 μg/ml polybrene, and 10 ng/ml IL4 and IL2. The cells were spun in a centrifuge for 1.5 h at 1100*g* at 32 °C. The cells were kept for another 3 h in an incubator at 32 °C. The cells were then kept at 37 °C overnight. The next morning, the media was replaced with fresh IMDM supplemented with IL4, βME, and 15 μM 4μ8c (or DMSO).

For RNA sequencing: 5 days after T cell activation, 5000 fluorescent (transduced) cells were FACS-sorted into 20-μl RLT buffer and frozen in − 80 °C. The RNA was extracted using 30 μl SPRI beads and eluted into 5-μl media of the following composition: 2 μl dNTP (10 μM), 2 μl Oligo-dT (100 μM), and 1 μl nuclease-free water. Four microliters of the elute was used as input into smart-seq 2 [39]. Pre-amplification was done using 8 PCR cycles. Library preparation was done using Nextera XT at 1/4 of the manufacturer-specified reaction size. The libraries were sequenced on an Illumina HiSeq 2500 50SE. The raw reads are deposited at ArrayExpression (E-MTAB-7104).

Reads were trimmed using Trimmomatic 0.36 using settings -phred33 ILLUMINACLIP:NexteraPE-PE.fa:2:30:10 LEADING:3 TRAILING:3 SLIDINGWINDOW:4:15 MINLEN:30. Reads were mapped using Kallisto 0.44.0 with settings -b 100 --single -l 180 -s 20. A custom R script collected the estimated counts into a total count matrix.

Differential expression analysis was performed using DESeq2. The control cells were compared to 4μ8c treated, with XBP1s overexpression, and with simultaneous 4μ8c treatment and overexpression. The heatmap shows the fold change of genes that in any comparison has an adjusted *p* value of 10^−10^. Other cut-offs yield similar results.

For cell proliferation assays, naïve cells were stained with CellTrace Violet following the protocol as described above.

For intracellular cytokine IL5 and IL13 detection, XBP1s- or empty vector-transduced cells were sorted by cell sorter (as described above for RNAseq), rested for two more days to propagate, and reactivated in CD3e/CD28-coated plates for 6 h. The cells were then stained with fixable live-dead dye, fluorescent dye-conjugated anti-IL13 and IL5 antibodies, and analyzed by FACS.

## Results and discussion

In this study, to understand the role of IRE1a-XBP1 pathway, our basic strategy was to use an in vitro Th2 differentiation model (Fig. 1b). Naïve T helper cells were activated by TCR activation in anti-CD3e/CD28-coated plates under Th2 differentiation condition for 72 h, rested for 42 h, and restimulated by TCR activation using anti-CD3e/CD28-coated plates. To perturb IRE1a-XBP1 pathway, we used a well-established drug 4μ8c that specifically blocks the pathway by inhibiting IRE1a endonuclease activity [40]. The drug was added to the culture media at 15-μM concentration at the beginning of the culture and during passage from the activation plate to the resting plate. The choice of drug concentration was determined by its highest IRE1a inhibition efficiency with lowest cell toxicity (Additional file 1: Figure S1). We compared transcriptomes of naïve and restimulated Th2 (drug treated and untreated) lymphocytes by RNA sequencing, identified XBP1 transcription factor binding sites in reactivated Th2 by ChIPmentation (ChIP-sequencing), and integrated the genome-wide data to predict direct targets and their regulatory role.

### T helper cells switch on the IRE1a-XBP1 pathway during in vitro activation

Activated and differentiated T helper cells secrete an abundance of cytokines. Therefore, a well-developed secretory machinery is a prerequisite for cells to adapt to this secretory stress. To predict the involvement of ER-stress/UPR pathway during T helper cell activation, we compared transcriptome of naïve and differentiated Th2 cells (restimulated Th2). Differentially expressed genes as obtained from this comparison were integrated in the “Protein Processing in the Endoplasmic Reticulum” KEGG pathway to visualize the components that are up- or downregulated. The analysis shows that when naïve T helper cells are activated and differentiated into Th2 cells, they upregulate expression of genes involved in the ER stress pathway (Additional file 1: Figure S2). Several factors that have previously been characterized as controllers of protein folding and secretion, including XBP1 itself, are upregulated during T helper cell differentiation.

To validate this prediction, and specifically investigate the involvement of the IRE1a-XBP1 pathway, we measured IRE1a mRNA and protein expression in Th2 lymphocytes differentiated and reactivated in vitro (Fig. 1b). The cells were analyzed by qPCR and Western blot to compare the mRNA and protein respectively. We found that both mRNA and protein level were upregulated in activated T helper cells (Fig. 1c, left and middle panel). It is known that phosphorylation of IRE1a denotes its functional state. We observed that the protein is phosphorylated in activated Th2 lymphocytes (Fig. 1c, right panel). This increased phospho-IRE1a can be explained by the increased synthesis of the protein, though we cannot exclude the possibility of increased kinase activity and auto-phosphorylation. The densitometric analysis of Western blot band suggests that both mechanisms, upregulation of protein synthesis and increased phosphorylation, are involved. Protein upregulation increased threefold, but the phospho-protein increased 4.5-fold (Fig. 1c).

Activated IRE1a splices the unspliced XBP1 (XBP1u) mRNA and produces a spliced XBP1 (XBP1s) mRNA isoform. We observed increases in the spliced form of XBP1 (XBP1s), both at mRNA and protein levels, upon T helper cell activation (Fig. 1d, e). Tunicamycin was used as a positive control. It is a drug that inhibits *N*-linked glycosylation and thereby causes accumulation of unfolded proteins (i.e., endoplasmic reticulum (ER) stress), and increases XBP1s by enhancing IRE1a activity. Specific inhibition of the IRE1a endonuclease activity by treating the cells with 4μ8c [40] abolished both the XBP1s mRNA and protein isoforms, confirming that the formation of the spliced form was dependent on IRE1a activity (Fig. 1d, e).

These results confirm that the IRE1a-XBP1 pathway is conserved in Th2 lymphocytes and upregulated during in vitro T helper cell activation. Next, we set out to investigate whether this also holds in vivo.

### In vivo activated T helper cells upregulate the IRE1a-XBP1 pathway

**Fig. 2:**
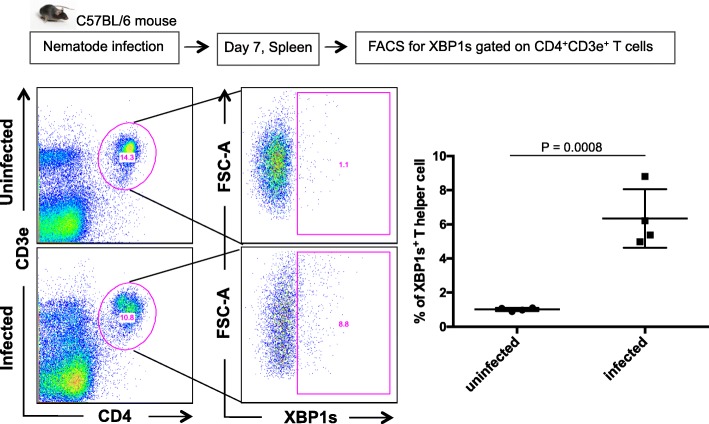
T helper cells upregulate IRE1a-XBP1 pathway in vivo during infection. Splenocytes from nematode (*Nippostrongylus brasiliensis*)-infected mouse (7 days post-infection) were stained with a PE-conjugated anti-XBP1s antibody and analyzed by flow cytometry (gating strategy: singlet > live cells > CD4^+^CD3e^+^ > XBP1s^+^). One representative FACS profile is displayed (left panel), and the graph containing all results (*n* = 4) is shown in the “right panel”

To test whether the IRE1a-XBP1 pathway is operational in CD4^+^ T cells in vivo, we infected C57BL/6 mice with the helminth parasite *Nippostrongylus brasiliensis*, a well-established model of Th2-driven immune responses [32, 41, 42]. After 7 days post-infection, we analyzed XBP1s protein expression in T helper cells by flow cytometry. We found T helper cells from worm-infected mice express significantly more XBP1s compared to uninfected control mice, suggesting an upregulation of the pathway (Fig. 2).

These results confirm that the pathway is active in vivo*.* Therefore, we set out to dissect the pathway using genome-wide approaches in Th2 lymphocytes.

### Genome-wide transcriptomic analysis of differential gene expression reveals IRE1a-XBP1-regulated genes

**Fig. 3:**
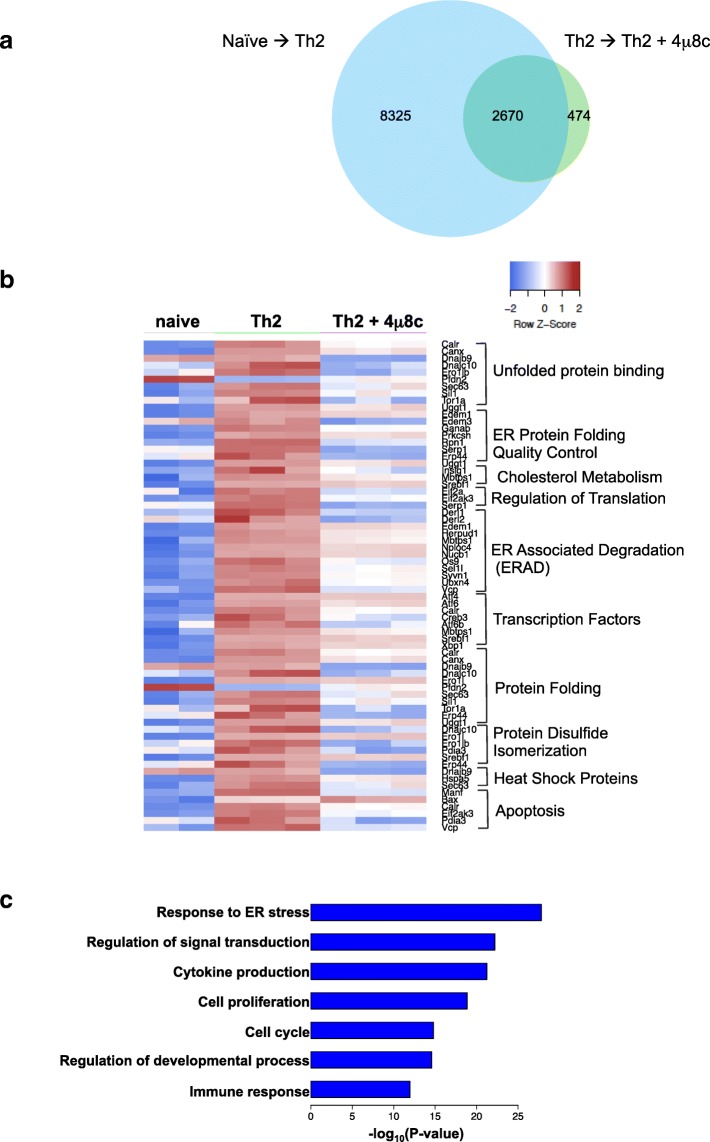
Differential gene expression in Th2 due to the inhibition of IRE1a-XBP1 by 4μ8c. Naïve T helper cells were activated under Th2 differentiation conditions in the presence or absence of 4μ8c. Cells were activated in anti-CD3e and anti-CD28 antibody-coated plates for 3 days, rested for 2 days, and reactivated in coated plates for 6 h. The RNAseq data were analyzed for differential gene expression. **a** Venn diagram showing the numbers of differentially expressed genes in different experimental conditions. “Naïve → Th2” indicates the differentially expressed genes between naïve T helpers and Th2 cells. “Th2 → Th2+4μ8c” indicates the differentially expressed genes between untreated and 4μ8c-treated Th2. **b** Heatmap showing differentially expressed genes that are well known to be involved in resolution of ER stress imposed by unfolded protein response. The heatmap shows scaled expression values denoted as row *Z*-score, in red-blue color scale with red indicating increased expression and blue indicating decreased expression. **c** Gene ontology (GO) analysis of the differentially expressed genes between Th2 and 4μ8c-treated Th2

To capture a global gene regulatory role of the IRE1a-XBP1 pathway, we compared in vitro activated Th2 cells to cells with inhibited IRE1a endonuclease activity by adding 4μ8c into the cell culture media. We then compared the transcriptomes of activated Th2 lymphocytes with or without inhibition of the IRE1a-XBP1 pathway. Transcriptomes of 4μ8c-treated and untreated Th2 cells were obtained by mRNA sequencing (RNA-seq). Quality control of the RNA sequencing data is shown in Additional file 1: Figure S3. Comparing transcriptomes of naïve and activated Th2 lymphocytes, we found that 10995 genes were differentially regulated upon Th2 activation. Inhibition of the IRE1a-XBP1 pathway by 4μ8c treatment resulted in differential expression of 3144 genes as compared to the untreated Th2 control (Fig. 3a, Additional file 1: Figure S3 right panel). Two thousand six hundred seventy of these genes were involved in Th2 differentiation (Fig. 3a). Hierarchical clustering of the genes reveals the groups of genes up- and downregulated upon 4μ8c treatment (Additional file 1: Figure S3, right). Detailed examination of these genes revealed many to be associated with the unfolded protein response and ER-stress, indicating a major impact of the IRE1a-XBP1 pathway (Fig. 3b) on these biological processes. The complete list of differentially expressed genes can be found in Additional file 2: Table S1. Gene Ontology (GO) analysis of these differentially expressed genes upon 4μ8c treatment to Th2 cells (i.e., IRE1a-XBP1 pathway regulated genes) showed that they are enriched in the following biological processes: “Response to ER stress” (GO:0006950), “Regulation of signal transduction” (GO:0009966), “Cytokine production” (GO:0001816), “cell proliferation” (GO:0008283), “cell cycle” (GO:0007049), and Immune response (GO:0006955) (Fig. 3c). These changes in the gene expression patterns upon IRE1a inhibition suggest extensive involvement of XBP1 transcription factor in Th2 activation and proliferation, as well as differentiation. Therefore, we set out to find the genome-wide chromatin occupancy patterns of the XBP1 transcription factor.

### XBP1 ChIPmentation reveals XBP1 direct target genes in Th2 cells

To identify the genome-wide chromatin occupancy of XBP1, we performed ChIPmentation, a recently developed method that has been shown to be faster, more sensitive, and robust than traditional ChIP-seq approaches [35], using a ChIP-grade antibody against XBP1. In vitro differentiated and reactivated Th2 cells were used for the XBP1 ChIP. Two independent biological replicates were performed. We obtained 19.3 million and 22.4 million pair-end reads for each replicate respectively. Using MACS2 [43] with a *q* value less than 0.01 and fold enrichment over 5, we identified 9031 and 7662 peaks, respectively, in the two replicates. Overlapping analysis using bedtools [44] suggested 5892 peaks were present in both replicates. Therefore, we only focused on these 5892 peaks for the downstream analysis.

**Fig. 4:**
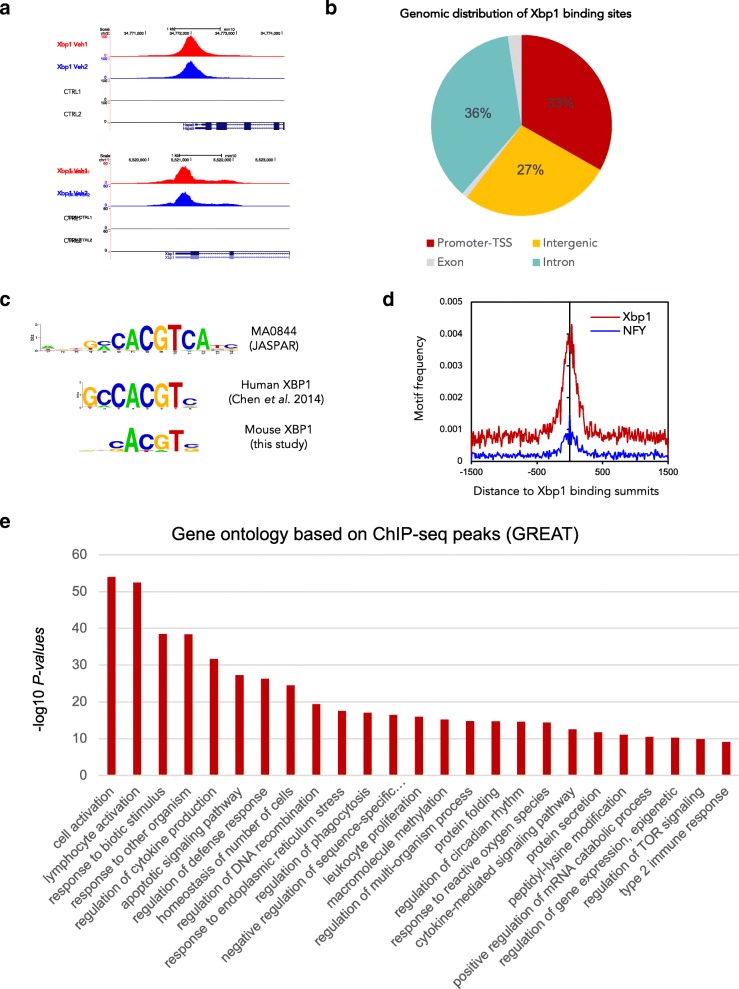
Genome-wide chromatin occupancy of XBP1 transcription factor in Th2 *lymphocyte*. XBP1 ChIPmentation was performed in in vitro differentiated Th2 cells to obtain genome-wide XBP1 chromatin occupancy. **a** Snapshot of XBP1 binding peaks around indicated representative genes from the UCSC genome browser. **b** Genomic distribution of XBP1 binding peaks. The sector corresponding to the promoter includes sequences up to 1 kb upstream and 100 bp downstream from the TSS. **c** Comparing the XBP1 motifs from the JASPAR database (top), ChIP-seq of the human breast cancer cell lines (middle), and mouse Th2 lymphocytes (bottom). **d** Motif frequencies of XPB1 and NF-Y around the binding peaks of XBP1. **e** Biological processes’ GO terms enriched within XBP1 binding peaks analyzed by GREAT

As expected, binding peaks were identified around promoter regions in known XBP1 target genes, such as *Hspa5* that encodes ER-chaperone protein BiP also known as Grp78; a binding event was also observed around the promoter of XBP1 itself (Fig. 4a), indicating potential auto-regulation of XBP1. To find out the genomic features associated with the XBP1 binding sites, we compared its peak location to the RefSeq genes using HOMER [40]. The majority of the XBP1 binding peaks were located within promoter (defined as upstream 1000 bp and downstream 500 bp relative to annotated transcriptional start sites) (36%) and intronic (35%) regions, and distal intergenic binding event (25%) were also frequently observed (Fig. 4b). The genomic distribution of XBP1 peaks indicates that it binds both promoters and potential enhancers.

To further characterize the XBP1 regulome, we performed de novo motif discovery using HOMER [45] to identify enriched DNA motifs within XBP1 binding regions. The top motif identified is the consensus sequence GCCACGT, which is almost identical to the human XBP1 binding motif defined in breast cancer cell lines (Fig. 4c) [46]. This indicates highly conserved binding specificities of XBP1 between human and mouse and across cell types. The top motif enriched in our mouse data also resembles the XBP1 motif from the JASPAR database [47], again supporting the high quality of our ChIPmentation data. The second most enriched motif is the NF-Y binding motif (Additional file 1: Figure S4C). Interestingly, the NF-Y motif has been frequently found around promoter regions of cell cycle genes, especially genes involved in G2/M cell cycle regulation [48, 49]. Both the XBP1 motif and the NF-Y motif co-occur around a subset of 258 XBP1 binding peaks (Fig. 4d), indicating potential cooperation between XBP1 and NF-Y transcription factors to regulate a subset of target genes. The list of target genes that are potentially co-regulated by XBP1 and NF-Y is displayed in Additional file 3: Table S2, and a complete list of XBP1 targets is also provided in Additional file 3: Table S2. The top five enriched motifs are displayed in Additional file 1: Figure S4C. To investigate the functions of XBP1-bound genes, we used GREAT [50] to characterize XBP1 binding peaks. Most of the significant GO terms are related to protein folding and ER-stress (Fig. 4e), which is consistent with the known biological role of XBP1.

Altogether, the ChIPmentation experiments predict a role of XBP1 in enhancing protein folding and secretion, as well as activation of Th2 lymphocytes.

### Integration of transcriptomic data and ChIP-seq data to unravel the XBP1-controlled gene regulatory network

**Fig. 5:**
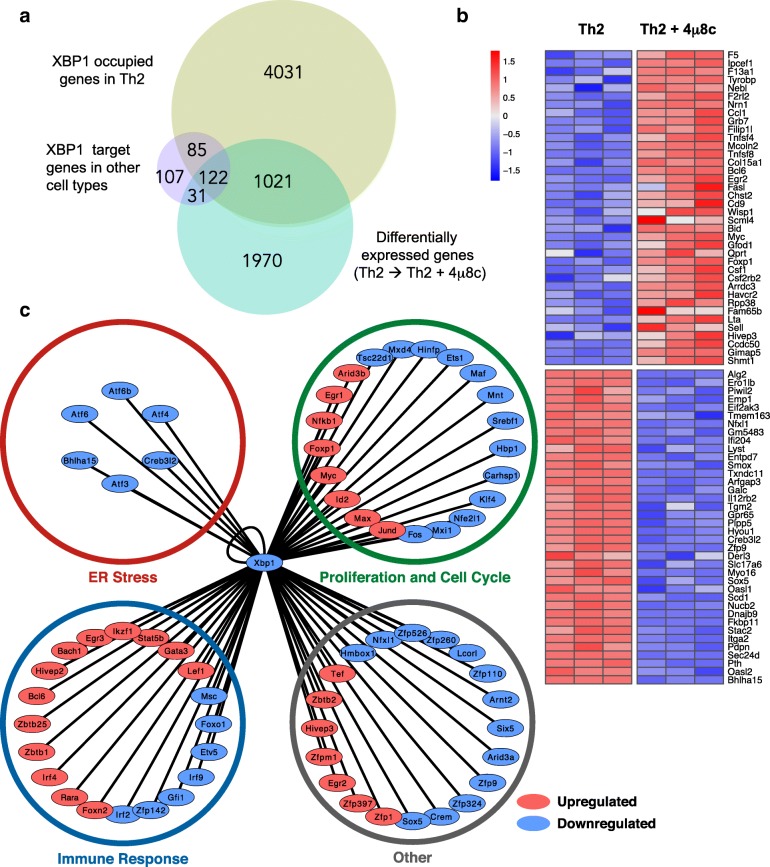
Integration of ChIPmentation and RNA-seq data reveals XBP1 direct target genes and its regulatory network. **a** Venn diagram comparing previously reported XBP1 target genes of other secretory cell types with the Th2 direct target genes of this study. XBP1 direct target genes of this study are those that are common in both “XBP1-occupied genes in Th2” and “Differentially expressed genes (Th2 → Th2+4μ8c)” categories. The XBP1 direct target genes of B cell/plasma cell, skeletal muscle cells, and pancreatic β-cells were as observed by Acosta-Alvear et al. [17] and have been used here for comparison. **b** Heatmap showing the pattern of XBP1 direct target gene expression. The top 38 genes that follow a distinct pattern have been displayed. **c** Transcriptional regulatory network: transcription factors that are direct target of XBP1. The genes in the network are differentially expressed (upregulated—red; downregulated—blue) up on 4μ8c treatment. The transcription factors that are not differentially expressed but have a XBP1 ChIPseq peak are shown in the right-hand side list

To reveal the XBP1-regulated direct target genes and its transcriptional regulatory network, we integrated the genome-wide transcriptomic data and ChIPmentation data. A direct target gene is defined by its differential expression upon IRE1a inhibition (i.e., 4μ8c treatment) and XBP1 transcription factor occupancy at the gene locus. We found 1143 direct target genes in Th2, of which 122 targets were previously reported as XBP1 direct target in other cell types (i.e., muscle, pancreatic β-cell, and plasma cell) (Fig. 5a). In this context, 1021 genes can be considered as Th2-specific. XBP1 action over its direct targets has no defined direction, containing genes up- and downregulated. The top 38 genes following either of these patterns are shown in Fig. 5b, and the complete list can be found in Additional file 4: Table S3. The most significant identified biological process and pathways are related to protein folding and ER-stress (Additional file 1: Figure S5), which are consistent with its known biological roles, and also include novel Th2-specific targets.

Despite the preponderance of XBP1’s role in controlling this pathway, other transcription factors are also found to be involved. To examine the regulatory cascade that follows XBP1 regulation, we built a transcriptional regulatory network by extracting annotated transcription factors with promoter or exonic/intronic ChIP-seq peaks (Fig. 5c). The complete list of the transcription factors can be found in the Additional file 5: Table S4. This network was further complemented by adding differentially expressed genes that have annotated interactions with the target transcription factors in the STRING database [37] (Additional file 6: Table S5).

The transcription factors that are directly regulated by XBP1 can be categorized into three broad functional categories involved in the following: resolution of protein secretory ER stress, regulation of cell cycle and proliferation, and controlling effector immune cell function. The ER-stress involved transcription factors are likely to facilitate cytokine secretion in Th2 lymphocyte. This prediction is based on the previous reports from secretory cells such as pancreatic acinar cells and plasma cells. These transcription factors, namely Bhlha15, Atf3, Atf6, Atf6b, Atf4, and Creb3l2, have been shown to be involved in secretory stress adaptation of the ER [9, 15, 51, 52].

The purpose of cell proliferation and cell cycle-related transcription factors could be to facilitate the controlled rapid expansion of activated Th2 cells. The immune response-related factors are likely involved in Th2 differentiation and cytokine production. Therefore, we wanted to test the effect of XBP1s downregulation in cytokine secretion, cell proliferation, and cytokine production.

### The IRE1a-XBP1 pathway controls cytokine secretion in T helper cells

**Fig. 6:**
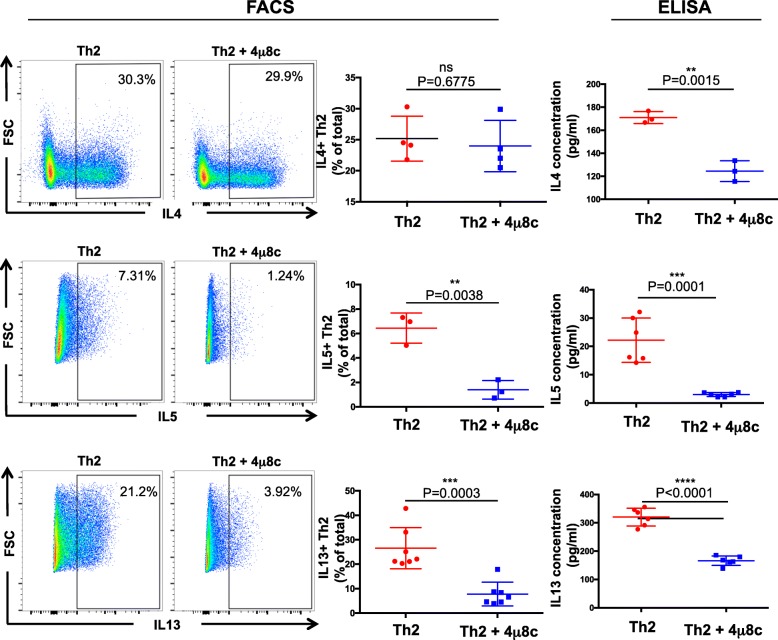
IRE1a-XBP1 pathway is required for cytokine expression and secretion in Th2 lymphocyte. Naïve T helper cells were cultured following Th2 activation condition in the presence of IRE1a inhibitor 4μ8c for 3 days, rested for 2 days, reactivated by coated plate, and analyzed by flow cytometry to detect intra-cellular cytokines IL4, IL5, and IL13 expression. Representative FACS profiles are displayed in the first two columns. The intra-cellular cytokine expression is compared in column 3, with three to seven independent biological replicates. Fourth column: cell culture supernatants from 4μ8c-treated or DMSO-treated Th2 were analyzed by ELISA to measure the cytokine concentration. FACS gating: lymphocytes > singlets > live cells > cytokines

The genome-wide comparison of XBP1s-regulated genes predicts that the factor is involved in secretion of cytokines. To validate this prediction, we blocked IRE1a endonuclease activity in Th2 cells and analyzed the cell culture supernatant to quantify the IL4 level by ELISA. We selected IL4 as a testable candidate cytokine because its mRNA and protein are unchanged by downregulation of XBP1 (Additional file 1: Figure S6A left panel, Fig. 6 left and middle panel of top row). We found that the secretion of IL4 is significantly inhibited in 4μ8c-treated cells (Fig. 6, right panel of top row). As expected, this result supports the involvement of the IRE1a-XBP1 pathway in facilitating cytokine secretion in Th2 cells as predicted. The inhibition of the pathway during restimulation phase has no significant inhibitory effect on IL4 secretion (Additional file 1: Figure S6B). This result suggests that the XBP1s is required during Th2 differentiation, possibly for the development of an efficient secretory machinery.

### The IRE1a-XBP1 pathway controls IL13 and IL5 cytokine expression

IL5 and IL13 are two prominent type 2 cytokines that are involved in eosinophilia, allergies and helminth infection. We found that inhibition of IRE1a-XBP1 pathway significantly suppresses the IL5 and IL13 protein expression and secretion into the culture medium (Fig. 6 right panels of middle and bottom row). Bioinformatics analysis of the Th2 transcriptome predicts that the IRE1a-XBP1 pathway positively controls IL5 and IL13 gene expression, because both the genes were identified as differentially expressed genes upon IRE1a inhibition (Additional file 2: Table S1). We validated this prediction by RT-qPCR-mediated gene expression analysis (Additional file 1: Figure S6A, middle and right panel) and flow cytometry (Fig. 6). These results suggest a transcriptional involvement of the pathway regulating IL5 and IL13. Notably, the IL4 mRNA and protein levels are not affected indicating specific regulation of IL5 and IL13.

### IRE1a-XBP1 pathway facilitates activation-dependent T helper cell proliferation

**Fig. 7:**
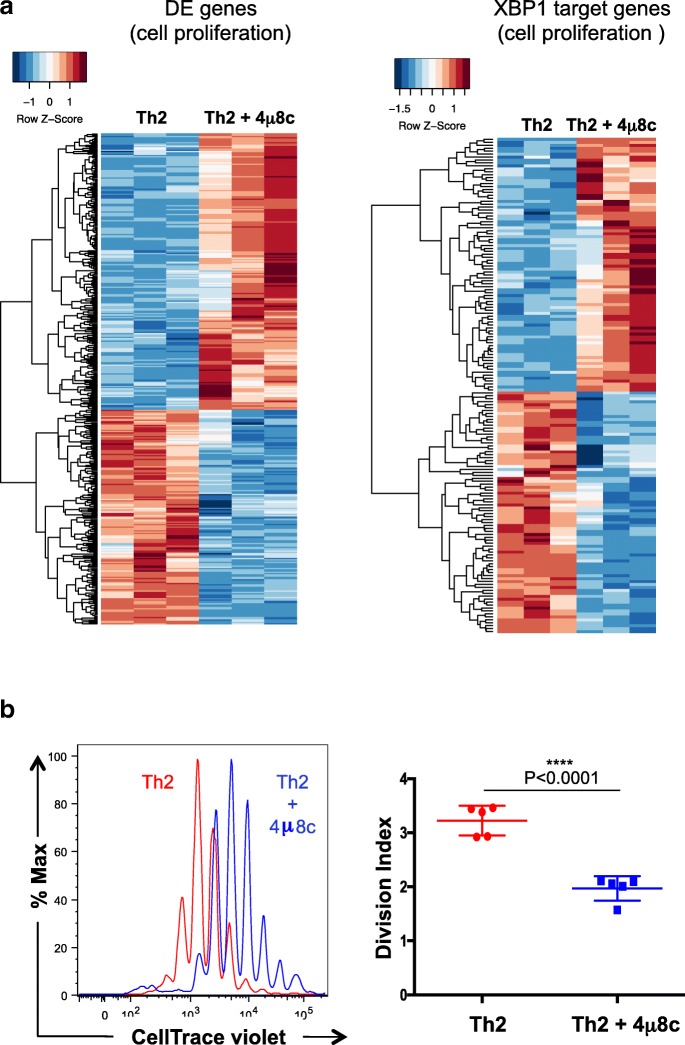
IRE1a-XBP1 pathway promotes activation-dependent Th2 cell proliferation and cell cycling. **a** Left panel: hierarchical clustering of differentially expressed cell proliferation-associated genes in the 4μ8c-treated and untreated Th2 transcriptome. Right panel: hierarchical clustering of XBP1 direct target genes that are known to be involved in cell proliferation. The heatmap shows scaled expression values denoted as row *Z*-score, in red-blue color scale with red indicating increased expression and blue indicating decreased expression. **b** Splenic naïve T helper cells were stained with CellTrace Violet dye and activated for 72 h under Th2 differentiation conditions and analyzed by flow cytometry. Generations of Th2 cells are in “red” and 4μ8c-treated cells are in “blue” in the histogram of cell proliferation (left panel, one representative experiment). Graphical representation of division index as obtained from five independent biological replicates (right panel)

Cell proliferation rate is a resultant outcome of positive and negative regulators’ interaction. We observed that genes encoding both positive and negative regulators of cell proliferation genes are differentially expressed when the IRE1a-XBP1 pathway was blocked by 4μ8c (Fig. 7a, left panel, Additional file 7: Table S6), of which many genes were found to be direct targets of XBP1 (Fig. 7a, right panel, Additional file 8: Table S7). This observation predicts a change in proliferation rate upon IRE1a inhibition. Therefore, we were interested in checking the effect of IRE1a-XBP1 inhibition on cell proliferation. We performed cell proliferation assay using Th2 cells. Naïve splenic CD4^+^ T cell were labeled with CellTrace violet and activated under Th2 differentiation condition in the presence or absence of 4μ8c. The fluorescent dye decay was monitored by flow cytometry. We found that downregulation of XBP1s inhibits cell proliferation significantly (Fig. 7b), but does not induce cell death (Additional file 1: Figure S7).

T helper cell proliferation is associated with differentiation and cytokine production. The reduced IL5 and IL13 expression (Fig. 6) could potentially be explained by the fact that cell proliferation is retarded. However, if reduced proliferation was the primary reason for lack of secretion, IL4 production would also be inhibited. Yet, we observed no significant change in IL4 expression upon IRE1a inhibition (Fig. 6, Additional file 1: Figure S6A). To examine this discrepancy further, we performed cell proliferation assays using IL13-GFP and IL4-GFP reporter mouse lines. In IL4-GFP expressing Th2 cells, we observed an inhibition of IL4 production in the first few generations of cell division up to 72 h upon 4μ8c treatment (Additional file 1: Figure S8). But at 96 h, the difference in IL4 expression becomes insignificant regardless of which generation of cell division the cells are in. This observation suggests that the retardation of proliferation due to the IRE1a inhibition is not sufficient to inhibit IL4 expression. In contrast, in IL13-GFP, we observed the decrease in IL13 expression from the very first generation and this continues throughout the later generations (Additional file 1: Figure S9).

### IRE1a inhibition delays cell cycle progression through the S and G2/M phase

**Fig. 8:**
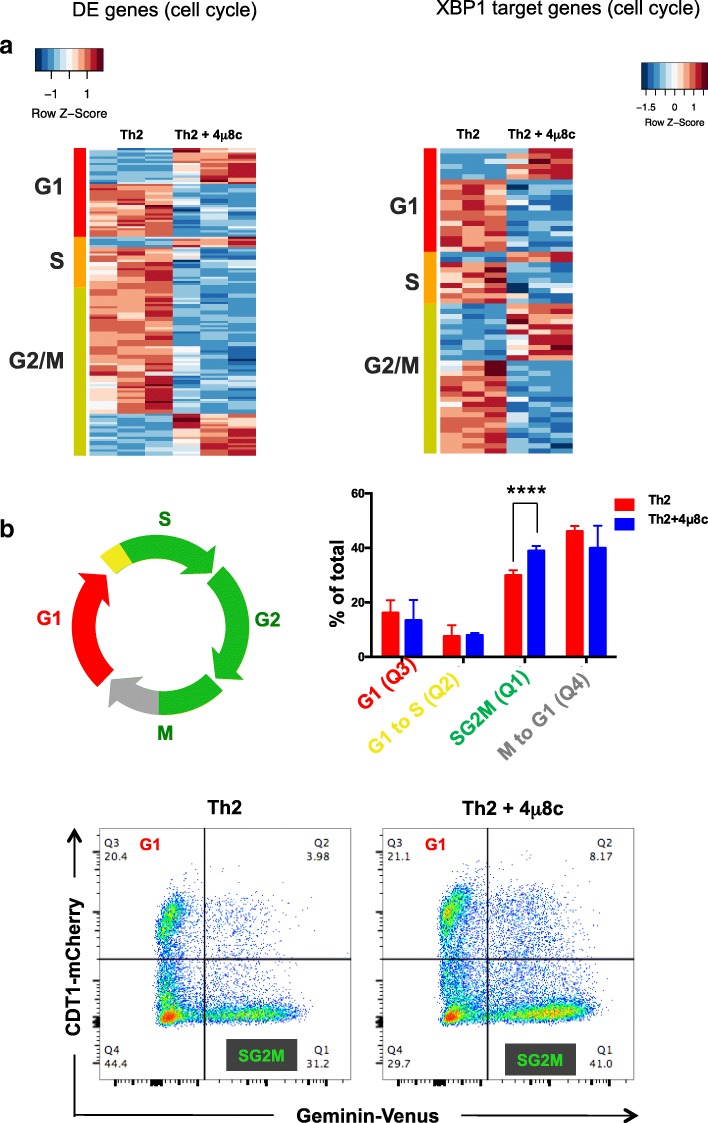
IRE1a inhibition delays cell cycle progression through the S and G2/M phase. **a** Left panel: heatmap of differentially expressed cell cycle stage-associated genes in the 4μ8c-treated and untreated Th2 transcriptome. Right panel: heatmap of XBP1 direct target genes that are known to be involved in cell cycling. The heatmap shows scaled expression values denoted as row *Z*-score, in red-blue color scale with red indicating increased expression and blue indicating decreased expression. **b** Cell cycle analysis of Th2 lymphocytes after 72 h of activation, using FUCCI mouse line that express mCherry-tagged CDT1 and Venus-tagged GEMININ. Upper left: diagrammatic representation of cell cycle stages in used FUCCI mouse. Upper right: comparison of cells (% of total) obtained from different stages of cell cycle in Th2 and 4μ8c-treated Th2 (*n* = 6). Lower panels: one representative FACS profile of Th2 and 4μ8c-treated Th2 showing CDT1 and GEMININ expressing cells

Bioinformatics analysis of differentially expressed genes (Th2 vs 4μ8c-treated Th2) and XBP1 direct target genes reveals several genes that are involved in controlling cell cycle progression through different stages (i.e., G1, S, G2/M) were clustered into two groups up- or downregulated (Fig. 8a). We took genes differentially expressed in 4μ8c-treated Th2 compared to untreated Th2 (adjusted *p* value < 0.05) (Fig. 8a, left, Additional file 9: Table S8) and the genes differentially expressed XBP1 direct target genes (Fig. 8a, right, Additional file 10: Table S9), and checked for known roles across distinct cell cycle stages using either a manually curated list based on RNA-seq data or published database [53]. We found many genes from all cell cycle stages (i.e., G1, S, and G2/M) were affected. To identify the cell cycle stages regulated by IRE1a-XBP1 pathway, we created and used a transgenic FUCCI (fluorescent ubiquitin cell cycle indicator) mouse strain that expresses mCherry-tagged Cdt1 and mVenus-tagged Geminin protein. The strain is similar to the one used in [31]. The G1 cells are mCherry+ mVenus− (Q3; Fig. 8b), G1-S cells are mCherry+ mVenus+ (Q2; Fig. 8b), and SG2M are mCherry− mVenus+ (Q1; Fig. 8b), while cells in mitosis and entering G1 are mCherry− mVenus− (Q4; Fig. 8b). We compared cell cycle profiles of vehicle and 4μ8c-treated Th2 cells during T cell activation. We found that cells accumulated in the S and/or G2/M phase when the IRE1a-XBP1 pathway is blocked (Fig. 8b). Similar results were obtained in a different approach using BrdU incorporation assay with DAPI staining (Additional file 1: Figure S10).

### Transgenic expression of XBP1s complements the 4μ8c-mediated inhibition of IRE1a endonuclease activity

To test whether the observed 4μ8c-treated phenotypes were due to the loss of XBP1s, we performed complementation assays by transducing a XBP1s expression vector into the Th2 cells in vitro. The vector encoded the spliced form of XBP1 (XBP1s), whose function is independent of IRE1a function. We found that stable ectopic expression of XBP1s negates the effect of 4μ8c treatment and there is no significant change in the transcriptome upon 4μ8c treatment when Th2 cells overexpress XBP1s (Additional file 1: Figure S11A). XBP1s overexpressing Th2 cells proliferate and differentiate normally in presence of 4μ8c (Additional file 1: Figure S11B and S11C respectively). These results strongly suggest that the phenotypes observed upon 4μ8c treatment are due to the loss of XBP1s.

## Conclusions

The primary aim of this study was to investigate the role of XBP1 transcription factor in Th2 lymphocytes and to identify the Th2-specific XBP1 target genes and their involvement regulating Th2 cell biology. We showed evidence that the IRE1a-XBP1 pathway is engaged in resolving secretory stress to meet robust cytokine synthesis and secretion, and controls multiple important cellular properties of T helper lymphocyte. It regulates activation-dependent T helper cell proliferation and cytokine production, the two key features of T helper cell during activation. The study revealed a large transcriptional regulatory network governed by XBP1. The comprehensive repertoire of XBP1-regulated genes and its genome-wide binding map provides a valuable resource for future work. We built a transcriptional regulatory map by integrating XBP1 ChIPmentation and RNAseq data, which portrays the bigger picture of the involvement of the XBP1 transcription factor in regulating target genes including other transcription factors. To visualize the data, we created an easily browsable online database available at http://data.teichlab.org.

ER-stress is known to be involved in several pathological situations. The pathway promotes cancer progression by providing metabolic advantage to the neoplastic cancer cells to acclimatize to the stressed tumor microenvironment. During the anti-tumor immune response, the XBP1 pathway induces tolerance in DCs. The pathway promotes asthmatic, allergic, and eosinophilic immune reactions and is involved in immunometabolism of macrophages in obesity. The pathway can be modulated by drug such as 4μ8c and STF-083010 and is under intensive investigation. Further studies will have to be carried out to determine whether the modulation of the pathway can bring patients’ benefit. This study shows evidence that perturbation of the IRE1a-XBP1 pathway may interfere with normal physiological activation of Th2 and could be exploited in settings where Th2 lymphocytes are pathologic such as asthma, allergies, and eosinophilia. Two prominent cytokines, IL5 and IL13, which promote allergies and eosinophilia, are under the control of IRE1a-XBP1 pathway in Th2 lymphocytes. In future, locus-specific mechanistic dissection of the XBP1-mediated transcription process in Th2 lymphocytes and in vivo immunobiological studies on novel Th2-specific XBP1 target genes are required to understand how the XBP1 transcription factor orchestrates locus control and to what extent it controls Th2-mediated immune responses.

## Additional files

Additional file 1: Supplementary figures S1-S11. (PDF 3129 kb)

Additional file 2: **Table S1.** List of differentially expressed genes upon IRE1a inhibition. (XLS 508 kb)

Additional file 3: **Table S2.** The list of XBP1 target genes and the genes that are potentially co-regulated by XBP1 and NF-Y. (XLSX 1208 kb)

Additional file 4: **Table S3.** List of XBP1 direct target genes as defined by its differential expression upon IRE1a inhibition (i.e., 4μ8c treatment) and XBP1 occupancy at the gene locus. (XLSX 433 kb)

Additional file 5: **Table S4.** List of transcription factors that are direct targets of XBP1. (XLSX 16 kb)

Additional file 6: **Table S5.** List of differentially expressed genes that have annotated interactions with the target transcription factors in the STRING database. (XLSX 24 kb)

Additional file 7: **Table S6.** List of differentially expressed genes encoding both positive and negative regulators of cell proliferation. (XLSX 48 kb)

Additional file 8: **Table S7.** List of XBP1 direct target genes that regulate cell proliferation. (XLS 21 kb)

Additional file 9: **Table S8.** List of differentially expressed genes that regulate cell cycle. (XLSX 45 kb)

Additional file 10: **Table S9.** List of XBP1 direct target genes that regulate cell cycle. (XLS 17 kb)

## Abbreviations

4μ8c: 7-Hydroxy-4-methyl-2-oxo-2H-1-benzopyran-8-carboxaldehyde
ChIP: Chromatin immunoprecipitation
DC: Dendritic cell
ELISA: Enzyme-linked immunosorbent assay
ER: Endoplasmic reticulum
FACS: Fluorescent-activated cell sorting
FUCCI mouse: Fluorescent ubiquitin cell cycle indicator mouse
IL: Interleukin
IRE1a: Inositol-requiring enzyme 1 alpha
Th: T helper
Th2: Type-2 T helper
UPR: Unfolded protein response
XBP1: X-box binding protein 1
XBP1s: Spliced form of X-box binding protein 1
XBP1u: Unspliced form of X-box binding protein 1
