# Supplementary material for: Genome-wide analyses reveal the IRE1a-XBP1 pathway promotes T helper cell differentiation by resolving secretory stress and accelerating proliferation
Source: Genome Med. 2018 Oct 24;10:76. doi: 10.1186/s13073-018-0589-3 (PMC6199730; doi:10.1186/s13073-018-0589-3)
Supplement: Supplementary file 1 — Supplementary figures S1-S11. (PDF 3129 kb) [file 13073_2018_589_MOESM1_ESM.pdf]

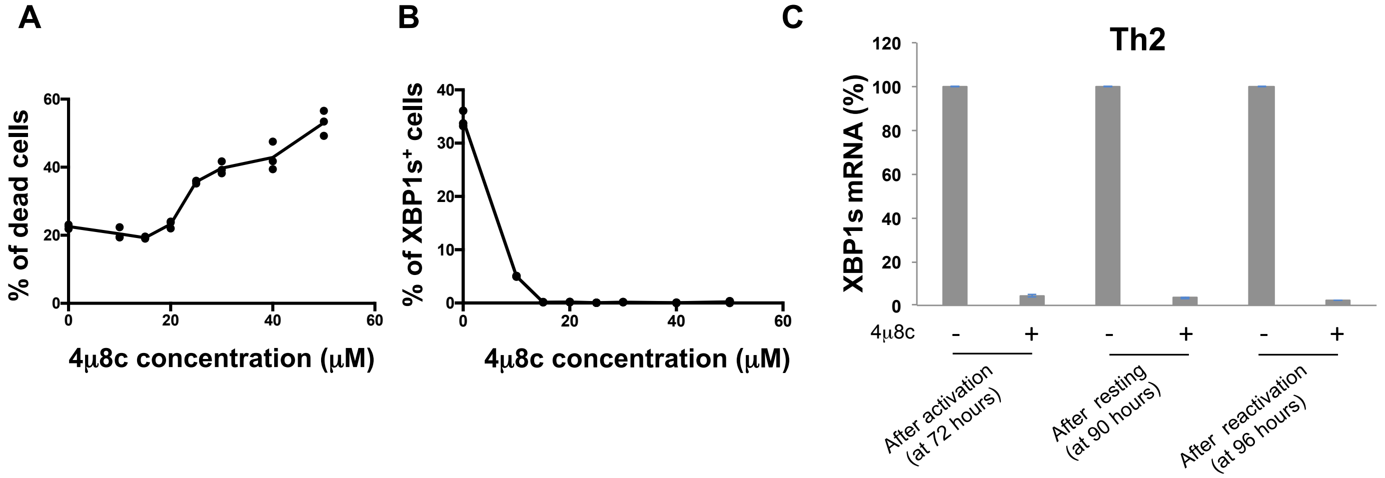

Figure S1. *Effect of 4μ8c treatment on Th2 lymphocytes*

A. Effect of 4μ8c treatment on cell viability. Viability of restimulated Th2 cells (4μ8c treated at different dosages and untreated) were measured by flow cytometry and compared. B. Inhibition efficiency of 4μ8c at different concentration. XBP1s expression in Th2 cells cultured in presence of different dosages of 4μ8c was measured by flow cytometry. C. Inhibition efficiency of 15 μM 4μ8c at different time-points of Th2 cell culture. Splenic naïve T helper cells were activated under Th2 differentiation condition in presence or absence of 15 μM 4μ8c. Cells were harvested at different time points and XBP1s mRNA expression was measured by qRT-PCR using XBP1s specific primer set that detect only the spliced form of XBP1.

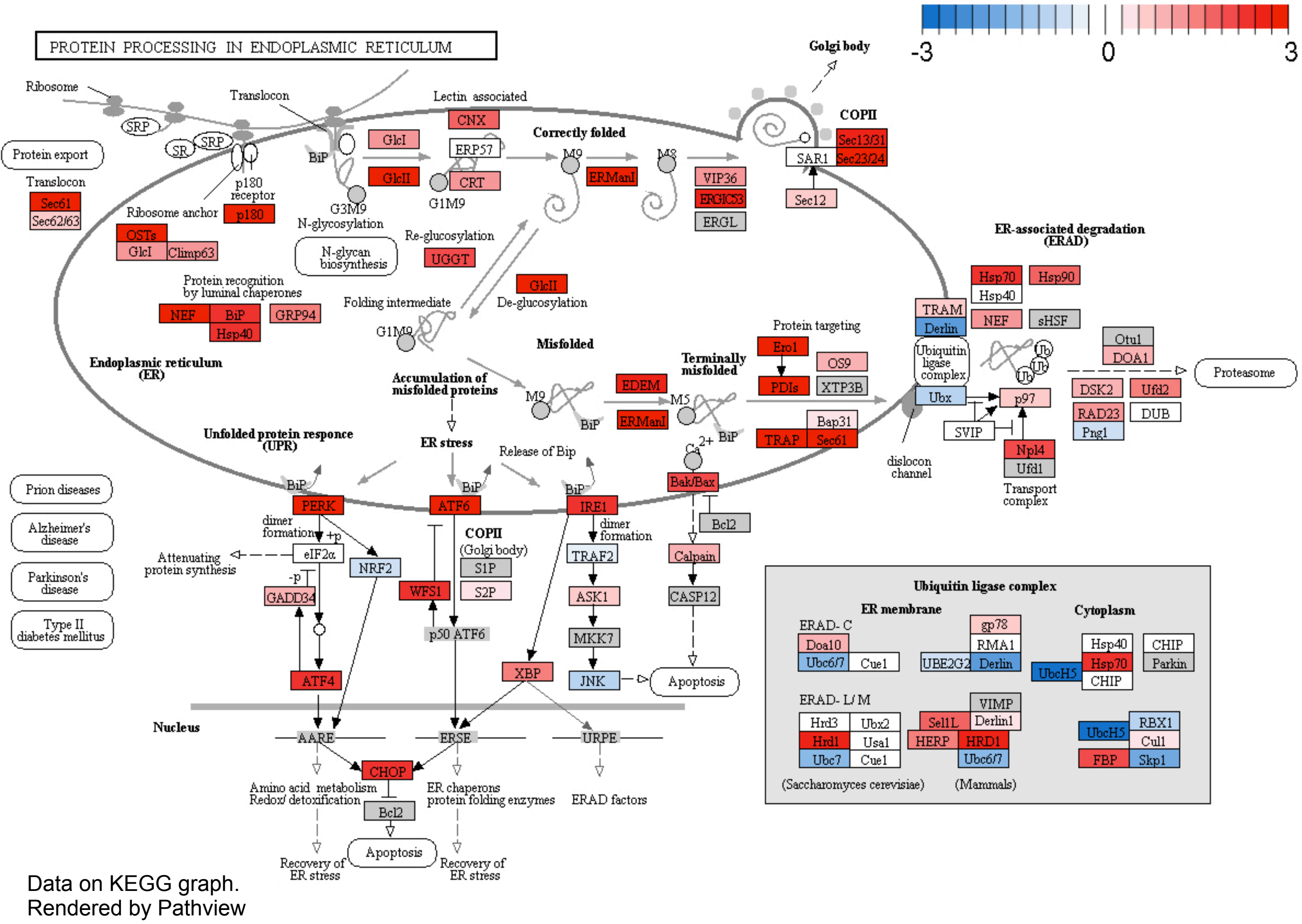

Figure S2. *Th2 lymphocytes upregulate the expression of genes that are involved in protein processing and folding in the endoplasmic reticulum.* Differentially expressed genes as obtained by comparing the transcriptome of naïve T helper and activated Th2 cells (log2 fold-change) were integrated in the "Protein Processing in the Endoplasmic Reticulum" KEGG pathway to visualize the components that are up or down regulated. "Red" represents upregulation and "Blue" represents down regulation of pathway genes. Rendered using the Pathview R package (Luo and Brouwer, 2013).

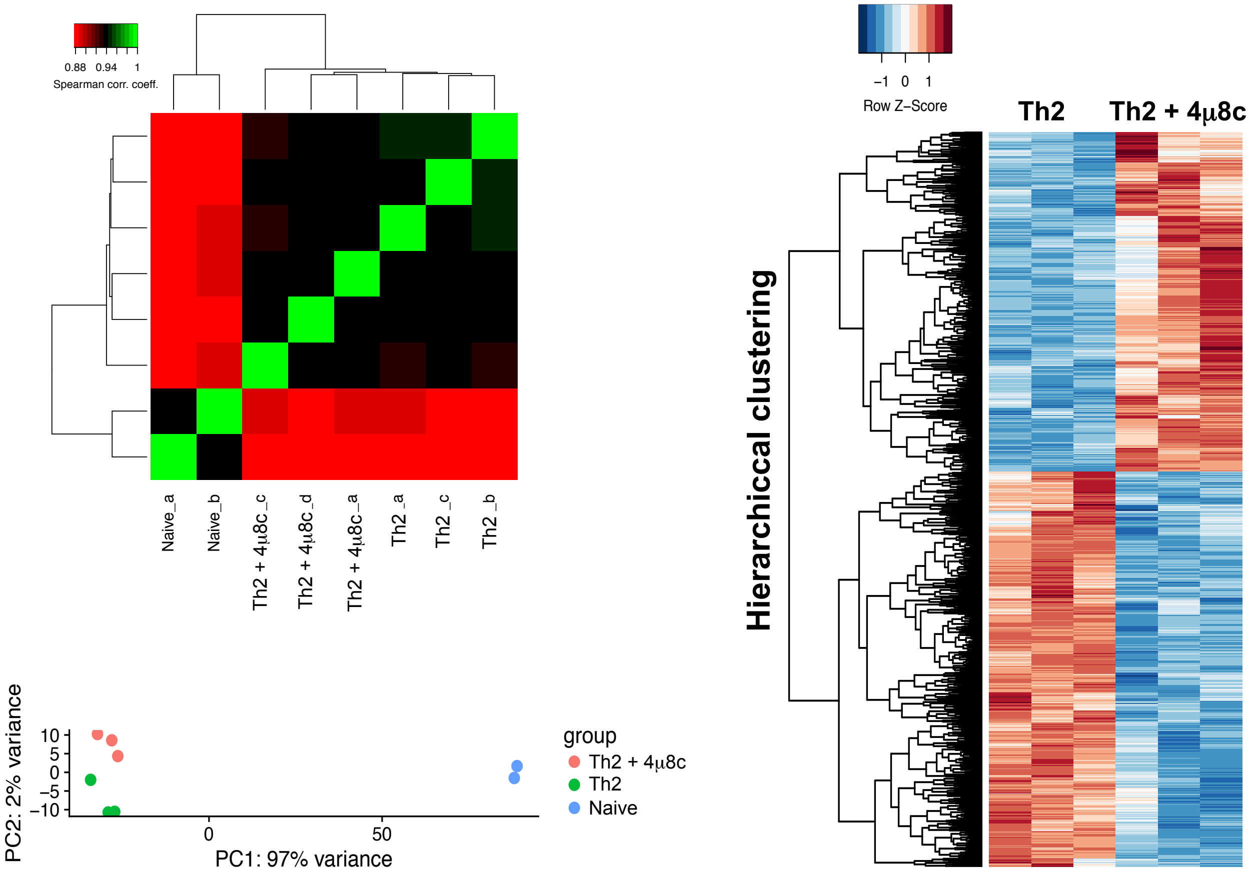

Figure S3. *Quality controls of transcriptomic data obtained from naïve, Th2 and 4μ8c treated and untreated Th2. Upper left panel:* Similarity between samples are shown based on Spearman correlation coefficients. Lower left panel: Principal component analysis. Plot of first two principal components for each sample calculated from regularised-log (rlog) transformed gene expression counts for all genes. Upper right panel: Hierarchical clustering of the genes in the RNA-seq data obtained from *in vitro* activated Th2 cells in presence or absence of IRE1a inhibitor 4μ8c. The heatmap shows scaled expression values denoted as row Z-score, in red-blue color scale with red indicating increased expression and blue indicating decreased expression.

A

	Replicate 1	Replicate 2
Peak number	9,031	7,662
Top 40% peaks overlap the other replicate	94.0%	96.5%

B

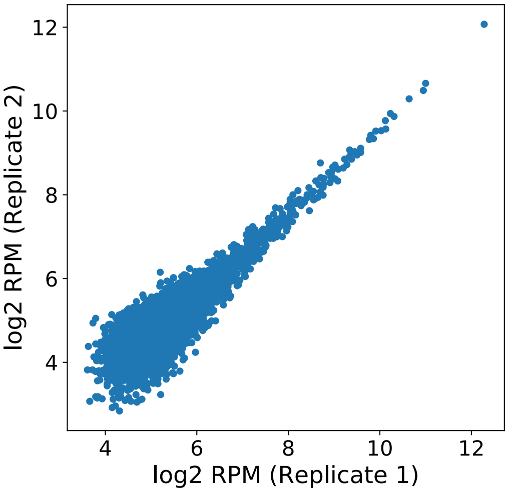

C

Rank	Motif	P-value	log P-pvalue	% of Targets	% of Background	
1	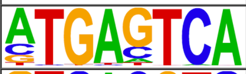	1e-385	-8.887e+02	25.37%	7.46%	**AP1**
2	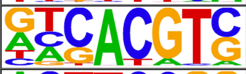	1e-347	-7.998e+02	35.71%	14.71%	**XBP1**
3	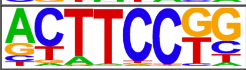	1e-191	-4.420e+02	40.04%	22.76%	**ETS**
4	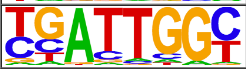	1e-138	-3.183e+02	12.56%	4.42%	**NFY**
5	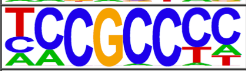	1e-90	-2.088e+02	25.12%	14.97%	**SP1**

Figure S4.

A. The number of peaks returned by MACS2 with q values less than 0.01 and fold enrichment over 5, and the overlapping statistics between the two replicates.

B. Correlation of the Xbp1 binding signals (log2 reads per million) between the two replicates, which shows excellent consistency.

C. Top 5 de novo motifs from XBP1 binding sites were analyzed by HOMER. Motifs are ranked by *p*-values (binomial test). "% of Target" shows the percentage of XBP1 binding sites containing the corresponding motif. "% of Background" shows the percentage of randomly selected background sequence containing the corresponding motif.

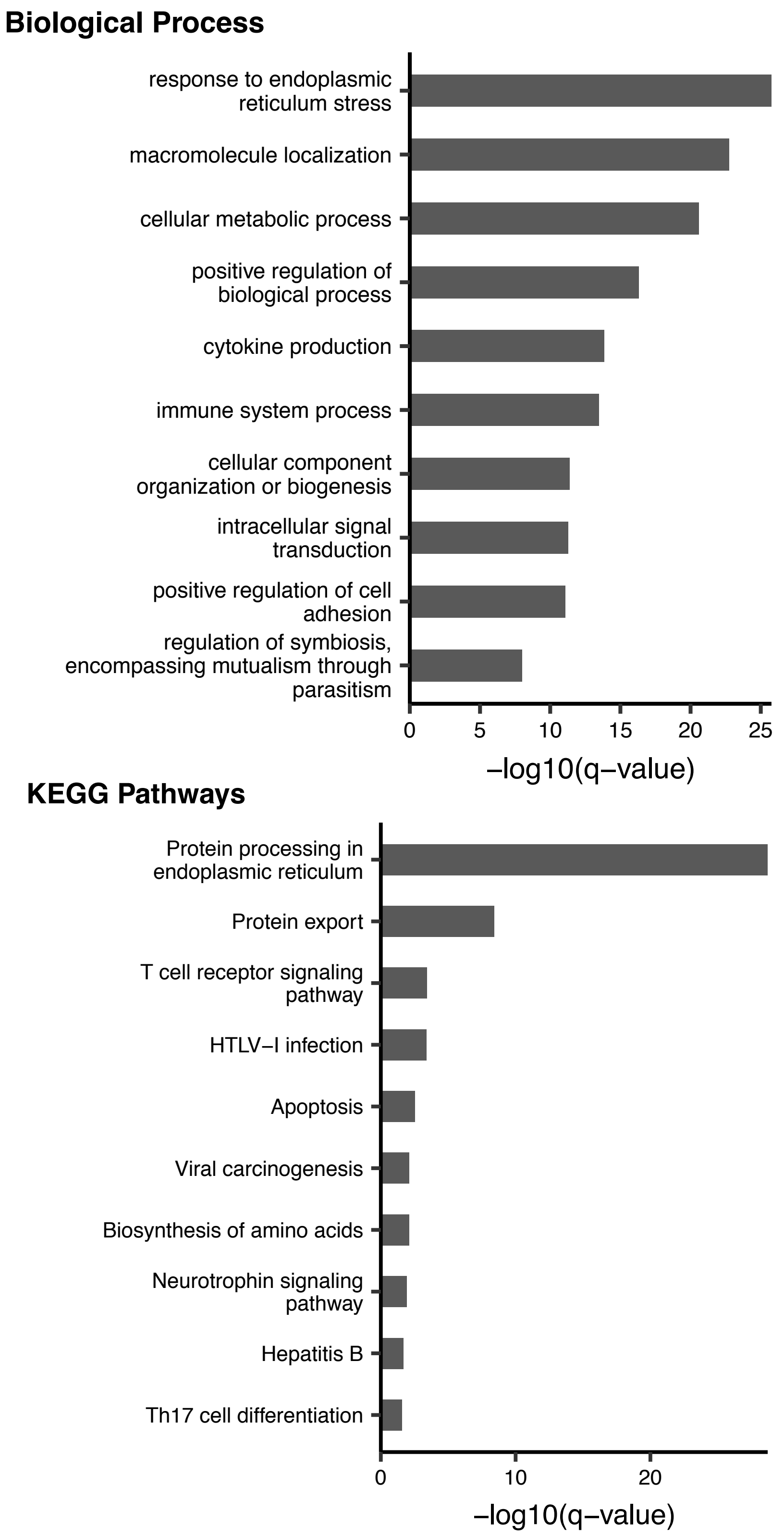

Figure S5. KEGG pathway and biological process analysis of XBP1 direct target genes.

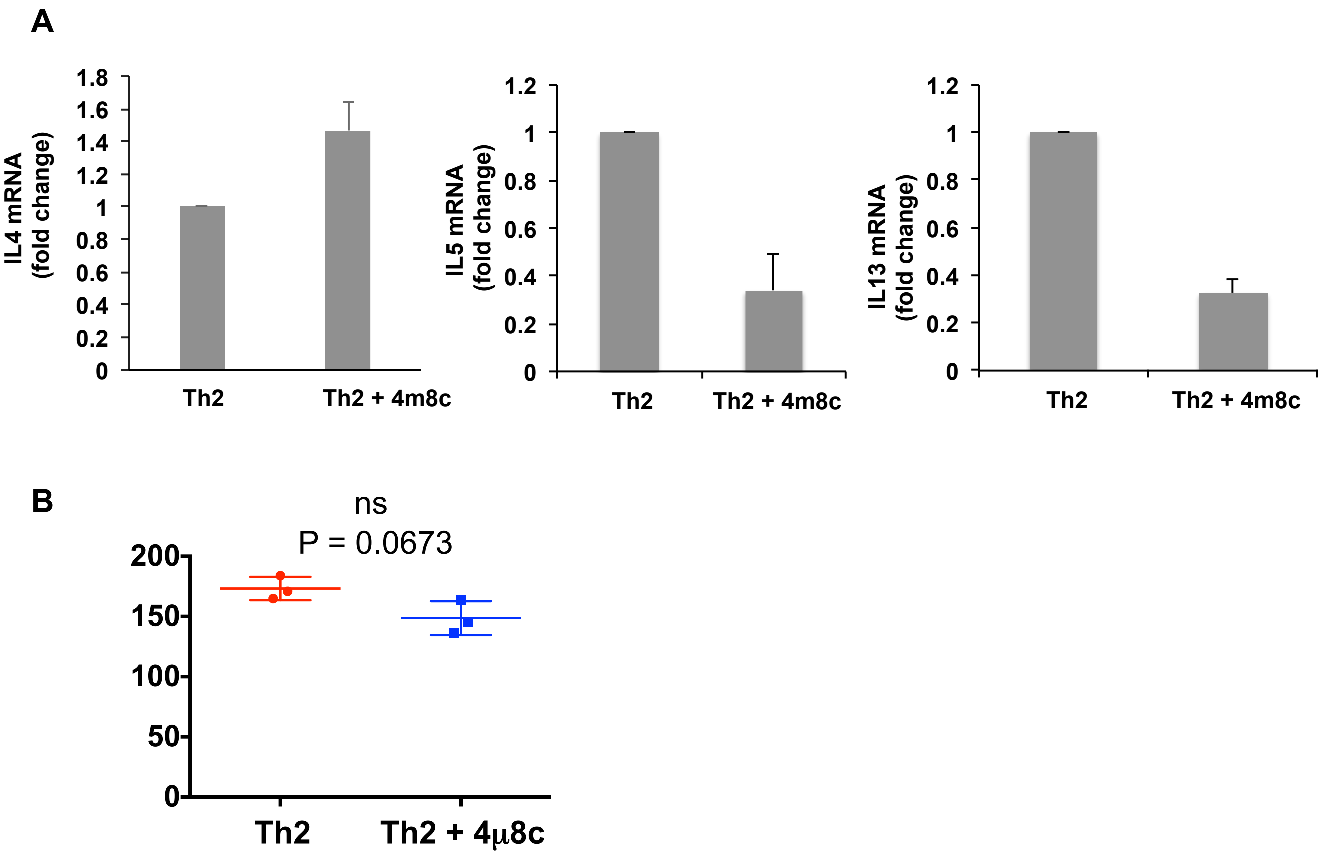

Figure S6. *A. Inhibition of IRE1a endonuclease activity reduces the expression of IL5 and IL13 mRNA but does not alter IL4 expression.* Naïve T cells were activated *in vitro* under Th2 differentiation conditions in the presence or absence of 4μ8c. mRNA expression was compared by qPCR. Expression level was normalized by the expression of Rplp0.

B. Naïve T helper cells were activated under Th2 differentiation condition in absence of IRE1a inhibitor 4μ8c for 3 days, rested for two days; and reactivated by anti-CD3e/CD28 antibody coated plate in presence of 4μ8c or DMSO (vehicle). Cell culture supernatants from 4μ8c treated or DMSO treated Th2 cells were analyzed by ELISA to measure IL4 concentration.

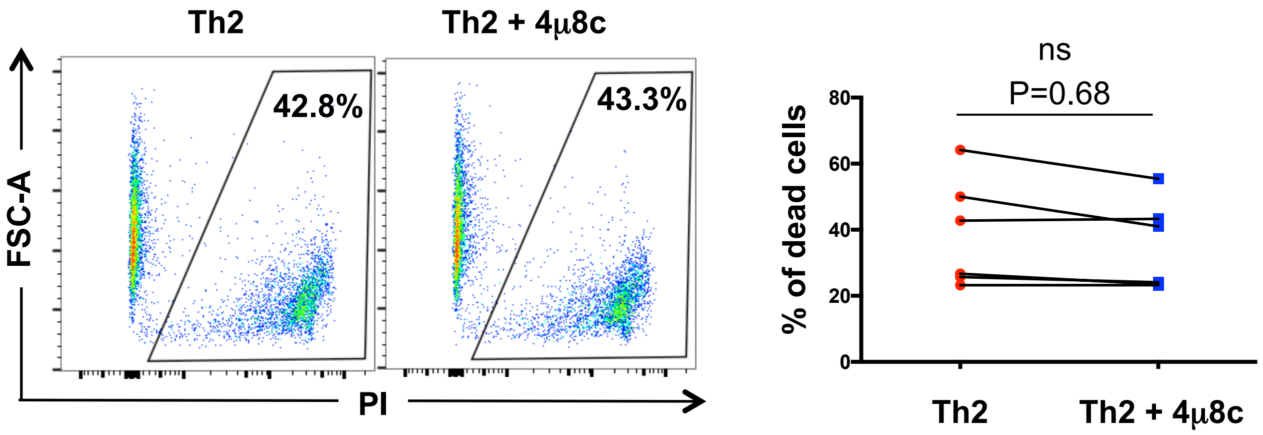

Figure S7. *Inhibition of IRE1a endonuclease activity does not alter Th2 cell viability.* Naïve T cells were activated *in vitro* under Th2 differentiation conditions in the presence or absence of 4μ8c, stained with propidium iodide, analyzed by flow cytometry and gated on singlets. Left panel: One representative flow cytometry result of five independent biological experiments is shown. Right panel: Graphical presentation of all five biological repeats.

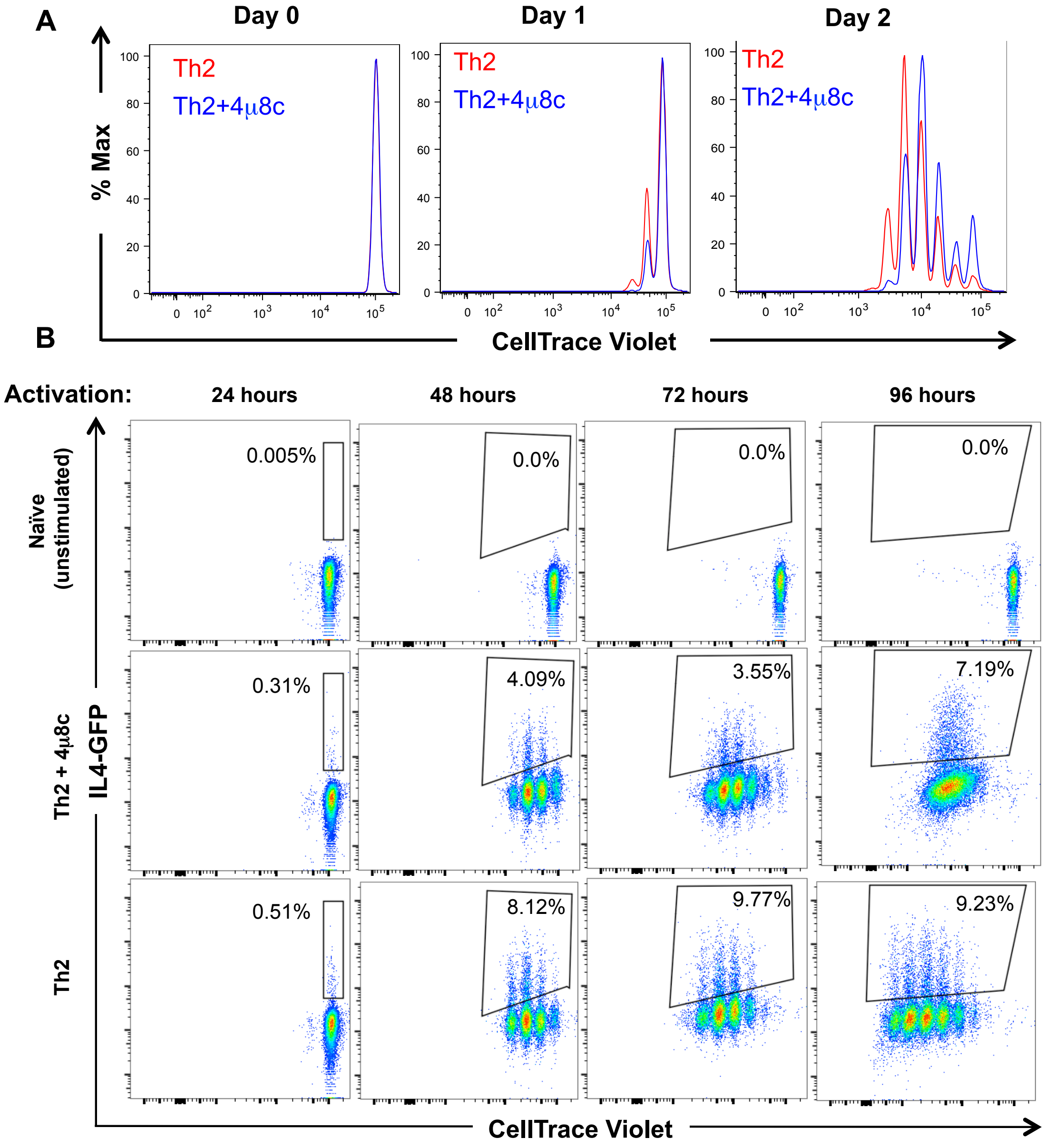

Figure S8. A. Detection of cell proliferation at early stages of Th2 activation. Splenic naïve T helper cells were purified from C57/BL6 mice, stained with CellTrace Violet, and activated up to 48 hours under Th2 differentiation conditions in presence or absence of 4μ8c. Cells from different time points were analyzed by flow cytometry. One representative result of three biological repeats is displayed. Gating strategy: Live cells > Singlet > CellTrace Violet. *B. Detection of cell proliferation associated IL4 expression:* Splenic naïve T helper cells were purified from IL4-eGFP mice, stained with CellTrace Violet, and activated up to 96 hours under Th2 differentiation conditions in presence or absence of 4μ8c. Naïve cells were maintained without activation. Cells from different time points were analyzed by flow cytometry. Gating strategy: Live cells > Singlet > CellTrace Violet, IL4-GFP. One representative result that is consistent with all aspects of four biological repeats is displayed.

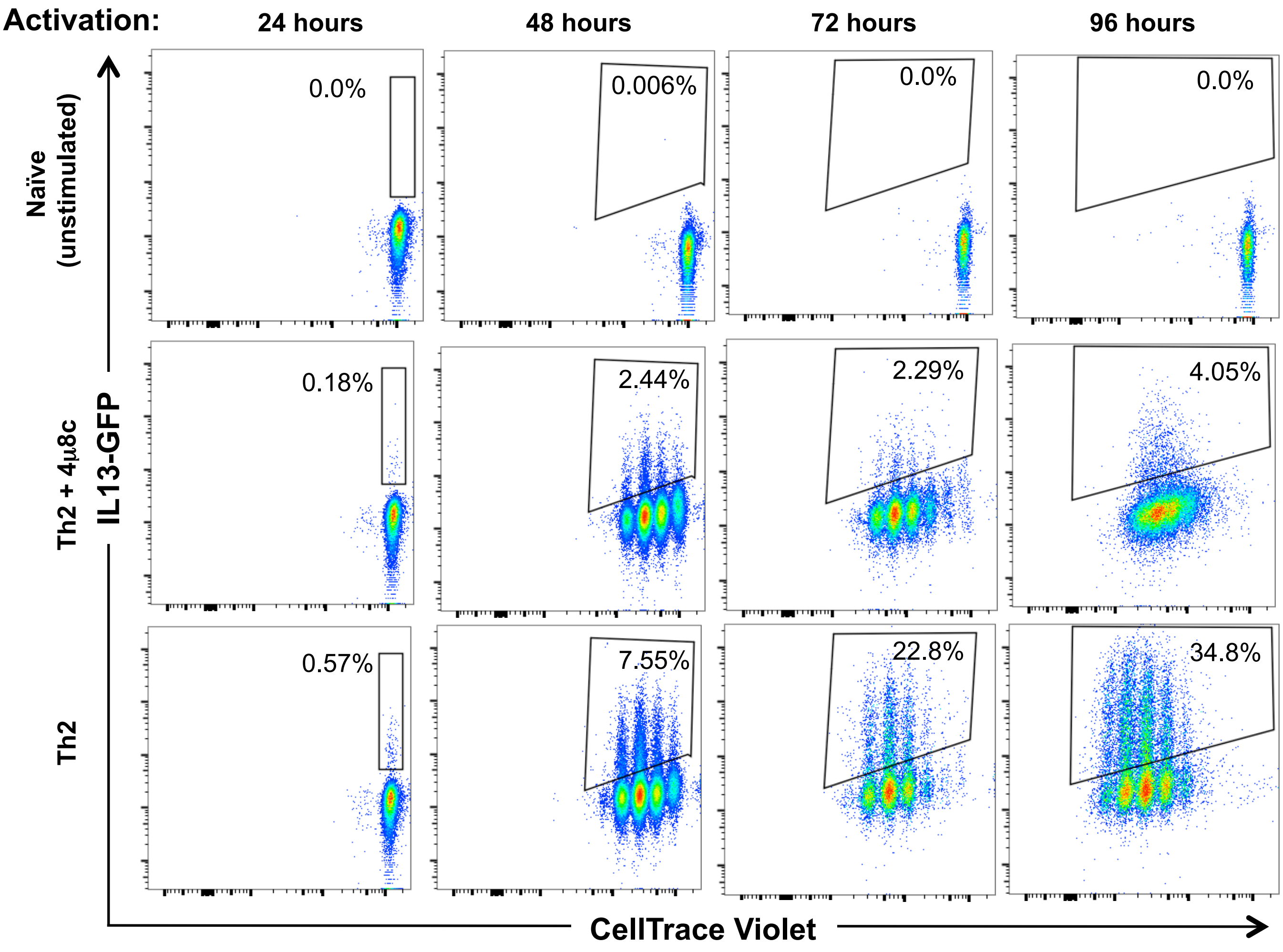

Figure S9. *Detection of cell proliferation associated IL13 expression using IL13-eGFP mouse due to IRE1a blockade:* Splenic naïve T helper cells were purified from IL13-eGFP mice, stained with CellTrace Violet, and activated up to 96 hours under Th2 differentiation condition in presence or absence of 4μ8c. Naïve cells were maintained without activation. Cells from different time points were analyzed by flow cytometry. Gating strategy: Live cells > Singlet > CellTrace Violet, IL13-GFP. One representative result that is consistent with all aspects of four biological repeats is displayed.

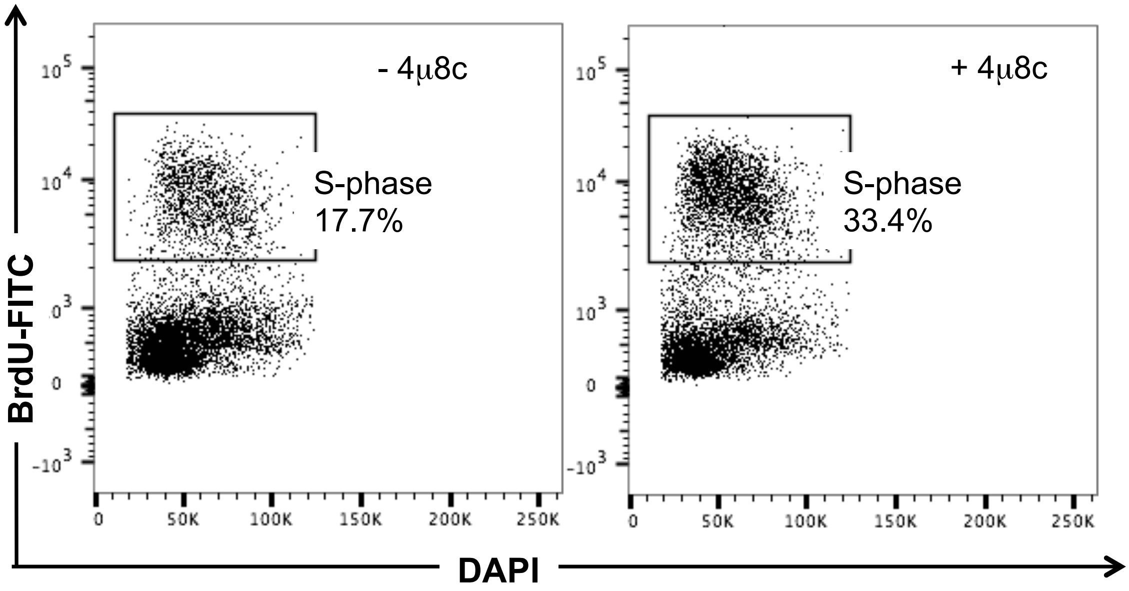

Figure S10. *Cell cycle analysis of Th2 lymphocytes using BrdU incorporation assay.* Splenic naïve T cells were activated in presence or absence of 4μ8c. BrdU incorporation was monitored by flow cytometry using FITC conjugated anti-BrdU antibody and DAPI-mediated DNA staining. One representative result that is consistent with all aspects of three independent biological replicates is shown.

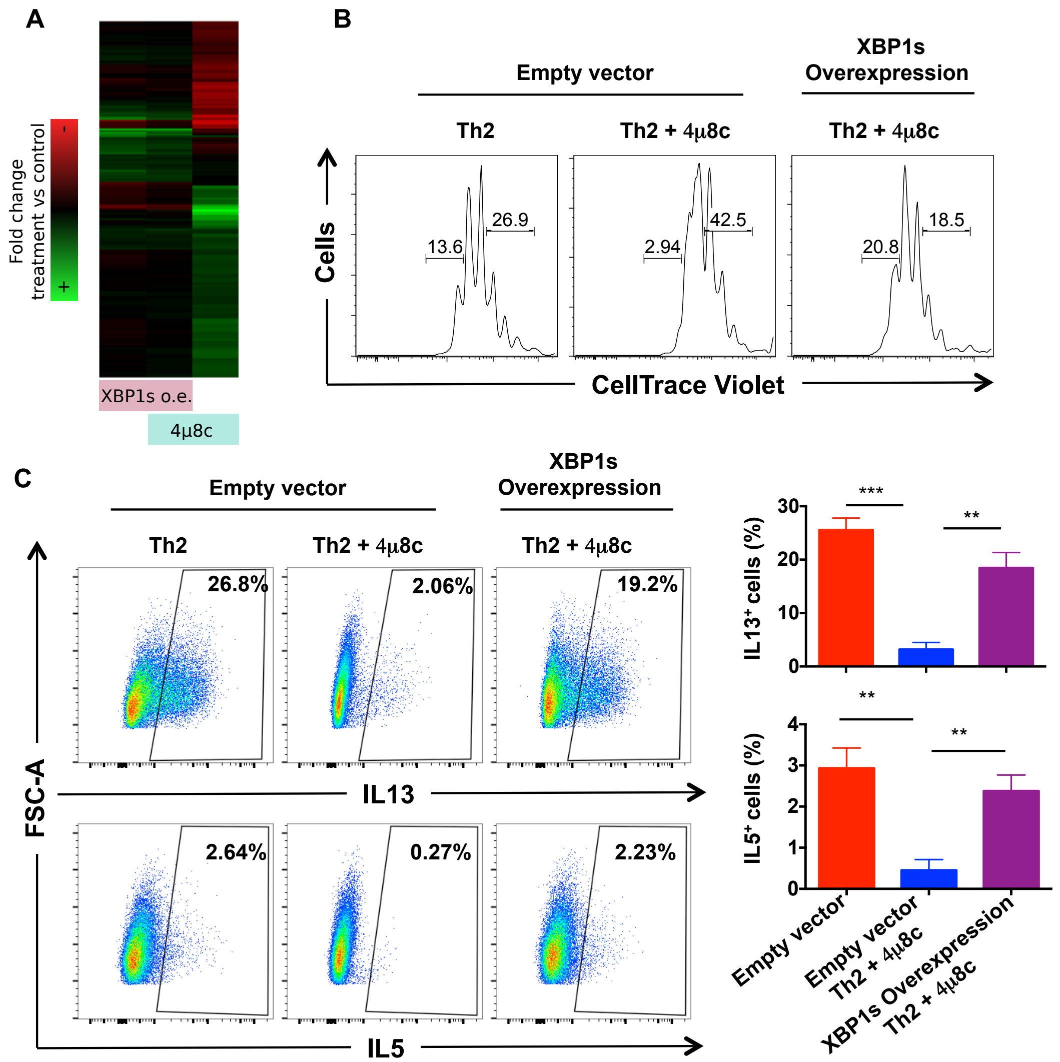

Figure S11. *Stable ectopic expression of XBP1s by viral transduction complements the 4μ8c-mediated inhibition of IRE1a endonuclease activity in Th2 cells.* Splenic naïve CD4+ T cells were activated *in vitro* under Th2 differentiation condition overnight in presence or absence of 4μ8c. The next day, cells were transduced with XBP1s expression vector or an empty vector, and cultured for 4 more days under Th2 differentiation conditions in the presence or absence of 4μ8c. Transduced cells were purified by cell sorting based on mCherry (*i.e.* vector-transduced cells) expression. A. Heatmap showing a comparison of transcriptomes in the presence or absence of 4μ8c (drug treatment) in Th2 cells stably overexpressing (o.e.) XBP1s or transduced with empty vector. This shows that 4μ8c has little to no impact in the presence of XBP1s o.e. B. Flow cytometric comparison of Th2 cell proliferation by fluorescent dye decay assay using CellTrace Violet in Th2 cells stably overexpressed (o.e.) XBP1s or transduced with empty vector and activated in presence or absence of 4μ8c. Gating: All cell > live cell > singlets > mCherry (to gate vector transduced cells) > CellTrace Violet. Data displayed is representative of three independent repeats with similar results. C. Flow cytometric comparison of IL13 and IL5 expression in Th2 cells stably overexpressing (o.e.) XBP1s or transduced with empty vector and differentiated in presence or absence of 4μ8c. Data displayed in the left panel is representative of three independent repeats with similar results. Graphical presentation of the average results is shown in the right panel.
